# Heat-Killed *Lacticaseibacillus paracasei* GMNL-653 Exerts Antiosteoporotic Effects by Restoring the Gut Microbiota Dysbiosis in Ovariectomized Mice

**DOI:** 10.3389/fnut.2022.804210

**Published:** 2022-02-04

**Authors:** Jhih-Hua Jhong, Wan-Hua Tsai, Li-Chan Yang, Chia-Hsuan Chou, Tzong-Yi Lee, Yao-Tsung Yeh, Cheng-Hsieh Huang, Yueh-Hsia Luo

**Affiliations:** ^1^Department of Computer Science and Engineering, Yuan Ze University, Taoyuan, Taiwan; ^2^Research and Development Department, GenMont Biotech Incorporation, Tainan, Taiwan; ^3^Department of Pharmacy, China Medical University, Taichung, Taiwan; ^4^School of Life and Health Sciences, The Chinese University of Hong Kong, Shenzhen, China; ^5^Warshel Institute for Computational Biology, The Chinese University of Hong Kong, Shenzhen, China; ^6^Aging and Diseases Prevention Research Center, Fooyin University, Kaohsiung, Taiwan; ^7^Biomed Analysis Center, Fooyin University Hospital, Pingtung, Taiwan; ^8^Ph.D. Program in Environmental and Occupational Medicine, Kaohsiung Medical University, Kaohsiung, Taiwan; ^9^Department of Life Sciences, National Central University, Taoyuan, Taiwan

**Keywords:** *Lacticaseibacillus paracasei*, gut microbiota, dysbiosis, whole-genome sequencing, gut-bone axis, IL-17A, RANKL (receptor activator for nuclear factor k B ligand), antiosteoporotic effects

## Abstract

Osteoporosis is a metabolic inflammatory disease, an imbalance occurs between bone resorption and formation, leading to bone loss. Anti-inflammatory diet is considered having the potential to ameliorate osteoporosis. Heat-killed probiotics exhibit health benefits in relation to their immunomodulatory effects, but the detail mechanism involved in gut microbiota balance, host metabolism, immunity, and bone homeostasis remains unclear. In this study, we evaluated the antiosteoporotic effects of heat-killed *Lacticaseibacillus paracasei* GMNL-653 *in vitro* and in ovariectomized (OVX) mice. Furthermore, whole-genome sequencing and comparative genomics analysis demonstrated potentially genes involved in antiosteoporotic activity. The GMNL-653 exerts anti-inflammatory activity which restored gut microbiota dysbiosis and maintained intestinal barrier integrity in the OVX mice. The levels of IL-17 and LPS in the sera decreased following GMNL-653 treatment compared with those of the vehicle control; mRNA levels of *RANKL* were reduced and *TGF-*β and *IL-10* enhanced in OVX-tibia tissue after treatment. The levels of IL-17 were significantly associated with gut microbiota dysbiosis. Gut microbial metagenomes were further analyzed by PICRUSt functional prediction, which reveal that GMNL-653 intervention influence in several host metabolic pathways. The analysis of whole-genome sequencing accompanied by comparative genomics on three *L. paracasei* strains revealed a set of GMNL-653 genes that are potentially involved in antiosteoporotic activity. Our findings validated antiosteoporotic activity of heat-killed GMNL-653 using *in vitro* and *in vivo* models, to whole-genome sequencing and identifying genes potentially involved in this gut microbiota–bone axis.

## Introduction

Environmental factors influence host health through gut microbiota (1). Gut microbial communities can tune host immunity, modulate gut endocrine function and neurological signaling, and produce vital metabolites that influence the host (2). Gut dysbiosis is associated with many diseases, including metabolic liver diseases, cardiometabolic diseases, obesity, and type 2 diabetes (1). Moreover, studies and emerging evidence have indicated that dysregulated gut microbiota correlate with decreased bone density, and inflammatory bowel disease (IBD) (3–5). A close relationship between bone health and gut microbiota exist that may involve in calcium absorption, bone mineralization, and immune signaling (6, 7). Osteoporosis, a common metabolic inflammatory disease, is characterized by low bone density and the destruction of bone tissue; an imbalance occurs between bone resorption and formation, leading to bone loss. Osteoporosis-induced fractures of the femur and vertebrae profoundly affect the life quality and mortality of aging populations, representing a serious public health problem, and incurring high medical care costs. Postmenopausal osteoporosis increases the expression of proinflammatory and osteogenic cytokines, including interleukin 1 (IL-1), interleukin 6 (IL-6), tumor necrosis factor-alpha (TNF-α), macrophage colony stimulating factor (MCSF), and the receptor activator of nuclear factor kappa-light-chain-enhancer of activated B cells ligand (RANKL) from osteoblasts, T cells, and B cells (8–11). Gut dysbiosis not only induces local or systemic inflammation but also dysregulates nutrients and calcium across the intestine and into systemic circulation. Several drugs (e.g., bisphosphonates, denosumab, teriparatide, and abaloparatide) are used to treat osteoporosis through the blocking of bone reabsorption, stimulating of bone formation, or both (12). Combined use of drugs and probiotic supplementation may represent a safe and potentially effective therapy strategy for osteoporosis.

Although probiotics are considered non-pathogenic live microorganisms, the safety profile of live probiotic supplement remains unclear in certain circumstances, particularly in regard to the translocation of bacteria from intestinal tissue to systemic circulation (13). Hence, the utilization of nonviable heat-killed probiotics has drawn increased attention. Several studies have reported that heat-killed probiotics exhibit health benefits in terms of their immunomodulatory effect (14, 15) in *in vitro* and *in vivo* models. Furthermore, inactivated *Lactobacillus* decreases the production of IgE in an ovalbumin (OVA) mice model (16), mediates the Th1/Th2 switch, and exhibits potential protective effects in food allergies (17). The structural components of probiotics, particularly the cell envelope, capsule, and cell wall components, may play a role in these immunological effects (18, 19). However, the possible mechanisms underlying the influence of heat-killed probiotics on gut microbiota balance, host metabolism, immunity, and bone homeostasis remain undetermined.

In the present study, we investigated the antiosteoporotic effects of three heat-killed *Lacticaseibacillus paracasei* (previously classified as *Lactobacillus paracasei*) strain GMNL-653 *in vitro* and in ovariectomized (OVX) mice. First, we observed that one *L. paracasei* GMNL-653 strain reduced lipopolysaccharide (LPS)-stimulated IL-6 and NO production in the mouse macrophage RAW264.7. Heat-killed GMNL-653 also increased calcium mineralization in human osteosarcoma MG63 cells mineralizing in culture and protected the OVX mice from bone loss, including increasing bone volume over tissue volume (BV/TV) and bone mineral density (BMD). Our results revealed that GMNL-653 restored ovariectomy-induced gut microbiota dysbiosis and maintained intestinal barrier integrity. Additionally, in the OVX mice, GMNL-653 treatment reduced the IL-17 and LPS levels compared with those of the vehicle control group, reduced the mRNA levels of *RANKL*, and increased the anti-inflammatory cytokines *TGF-*β and *IL-10* in tibia tissue. We applied the whole-genome sequencing technique and comparative genomics analysis to further examine these three *L. paracasei*. Our results indicated that the genes related to carbohydrate transport/metabolism and the cell wall/membrane/envelope biogenesis of GMNL-653 are worthy of future investigation. In summary, our results revealed that heat-killed GMNL-653 exhibited antiosteoporotic activity through the gut microbiota–bone axis.

## Experimental Procedures

### Bacterial Culture and Heat-Inactive Preparations

*Lactobacilli* were cultured in 1 mL of MRS broth at 37°C for 20 h, and subcultured at a 1:100 ratio in fresh MRS medium. After 20 h of subculture, the bacteria were centrifuged at 13,000 rpm for 1 min, and the bacterial pellet was then washed twice with PBS. The bacteria were suspended in PBS, with the bacterial concentration adjusted to 1 × 10^10^ cells/mL. The heat-killing method was performed at 90°C for 30 min or through autoclaving at 121°C for 15 min. *Lactobacilli* used in this study were *Limosilactobacillus reuteri* (previously classified as *Lactobacillus reuteri*; CCTCC M 209263), *Lacticaseibacillus rhamnosus* (CCTCC M 203098), *Lacticaseibacillus salivarius* (GMNL-678), and five *Lacticaseibacillus. paracasei* strains (#1:CCTCC M 204012; #2: CCTCC M 2011331; #3: GMNL-653/BCRC-910721; #4: GMNL-855; and #5: BCRC-16100/ATCC 11582) were provided by GenMont Biotech (Tainan, Taiwan).

### Cell Culture

The RAW 264.7 cells were cultured in Dulbecco's modified Eagle's complete medium (DMEM) and supplemented with 10% fetal bovine serum (FBS; Invitrogen, MA, Unites States) and 20 μg/mL gentamicin. The cells were seeded with 4 × 10^5^ cells/well in a 24-well plate and then incubated in a humidified atmosphere at 37°C with 5% CO_2_. The supernatant was removed, and the adhered cells were washed twice with sterile PBS. Next, fresh DMEM without FBS was added for 2 h of starvation. The cells were treated with or without heat-killed bacteria for 2 h, and then LPS (*Escherichia coli* O111:B4; SI-L4391, Sigma-Aldrich, St. Louis, MO, United States) was added to yield a final concentration of 100 ng/mL for a further 20 h of incubation. The supernatant was collected for cytokine and NO measurement.

### *In vitro* Model for Calcium Mineralization

Mineralization was induced on confluent monolayers through the addition of osteogenic culture medium (OIM) containing 10% (v/v) FBS, 10 mM glycerol-phosphate, 100 mM dexamethasone, and 0.05 mM vitamin C. Human osteosarcoma osteoblastic MG63 cells were cultured in a 24-well plate (3 × 10^4^ cells/well) with DMEM containing 10% FBS. Heat-killed GMNL-653 were adjusted to an appropriate density using OIM medium, and MG63 cells were added. Alizarin red S (ARS) staining was employed to evaluate calcium deposits. The cultures were incubated at 37°C with 5% CO_2_ with changes of OIM medium every 7 days. After 28 days, the cells were washed twice with PBS and then fixed with 70% acetic acid for 1 h. The cells were stained with 2% ARS (pH 4.1–4.5) at room temperature for 30 min with gentle shaking. Following the removal of the ARS dye, the cells were washed with H_2_O five times. Excess water was removed from the plates, which were then stored at −20°C prior to dye extraction. For quantification of the ARS staining, 200 μL of 10% (v/v) acetic acid was added to each well in the 24-well plate, and the plate was incubated at room temperature for 30 min with gentle shaking. The cells were then scraped from the plate and transferred into a 1.5-mL microcentrifuge tube. After being vortexed for 30 s, the tubes were heated to 85°C for 10 min and then placed into ice for 5 min. The tubes were centrifuged at 20,000 g for 15 min, and 200 μL of the supernatant was transferred into a new 1.5-mL microcentrifuge tube. Next, 75 μL of 10% (v/v) ammonium hydroxide was added to neutralize the acid. Aliquots (50 μL) of the supernatant and standard were measured at 405 nm in a 96-well format.

### Enzyme-Linked Immunosorbent Assay

Levels of IL-6 and interleukin 17 (IL-17A) were measured through enzyme-linked immunosorbent assay (BioLegend) according the instructions of the manufacturer. Sera LPS was detected using a Pierce LAL Chromogenic Endotoxin Quantitation Kit (Thermo Scientific, MA, United State).

### Animals

Twenty-four female ICR mice aged 8 to 10 weeks were housed in standard cages, maintained under specific pathogen-free conditions, and fed sterilized food and autoclaved water. The mice were either OVX or sham-operated with an intraperitoneal injection of anesthetic following standard protocol and were divided into three categories with eight mice in each group (sham, OVX + H_2_O, and OVX + GMNL-653). Mice in OVX + GMNL653 received GMNL-653 treatment once per day after the OVX or sham surgery for 28 days. At the end of experiment (4 weeks), the animals were sacrificed for further analysis.

### Micro-Computed Tomography Measures

The alterations in trabecular site of tibia were determined using a high resolution tomography image system micro-CT (SkyScan 1076, kontizh, Belgiumm resolution 18 μm per slice). After positioning right tibia, parameters such as total volume (TV, mm^3^), bone volume/total volume (BV/TV, %), apparent density refer to bone mineral density (BMD) (mgHA·ccm-1) were scanned. The locations of analysis were selected under proximal to the growth plate 100 slices and excluded cortical bone.

### RNA Isolation and Quantitative Real-Time Reverse Transcription-Polymerase Chain Reaction

The tibia from the OVX and sham mice were dissected, and the soft tissue was removed. The sample was pulverized using liquid nitrogen, and TRIzol reagent was added for RNA isolation. The purified RNA concentration was measured and stored at −80°C, and cDNA was synthesized with the total RNA (5 μg). Quantitative real-time reverse transcription-polymerase chain reaction (PCR) was used to measure *TGF-*β, *RANKL, IL-10, bone morphogenetic proteins 2 (BMP2), tartrate-resistant acid phosphatase 5b (Trap-5)*, and *glyceraldehyde-3-phosphate dehydrogenase (GAPDH)* expression, with the assays performed using 2 × Rotor-Gene SYBR Green PCR Master Mix (204076; QIAGEN). The reaction mixtures were prepared by mixing aliquots of cDNA, 3 μL of forward and reverse primer, and 5 μL of 2 × Rotor-Gene SYBR Green PCR Master Mix to obtain a final volume of 10 μL. The reaction mixtures were analyzed on a Rotor-Gene Q 2plex system. Quantitative values were obtained from the threshold cycle (Ct) number. The relative mRNA levels of the target genes were derived using the equation 2 – ΔCt, where ΔCt is Ct_targetgene_ − Ct_GAPDH_. Data were presented as the fold relative to the control value.

### 16S Ribosomal RNA Gene Amplicon Sequencing

Fecal samples were collected after 28 days of GMNL-653 treatment, and all specimens were extracted using a QIAGEN DNA kit according to the manufacturer's instructions. DNA samples were analyzed with a 260/280 OD in the range of 1.8 to 2.0. 16S ribosomal RNA (rRNA) PCR was performed using metagenomic DNA as a template, which was amplified with the bacterial-specific primers S17 (5′-TCG TCG GCA GCG TCA GAT GTG TAT AAG AGA CAG CCT ACG GGN GGC WGC AG-3′) and A21 (5′-GTC TCG TGG GCT CGG AGA TGT GTA TAA GAG ACA GGA CTA CHV GGG TAT CTA ATC C-3′). The amplified DNA sizing was verified using a fragment analyzer (5300; Agilent Technologies), and sequencing was conducted using a Illumina MiSeq platform. DNA samples were assigned indices and Illumina sequencing adapters with the Nextera XT Index Kit v2. After library construction, the samples were mixed using a 600-cycle MiSeq Reagent Kit v3 at a final concentration of 4 pM, loaded onto a MiSeq cartridge, and then transferred onto the instrument. Automated cluster generation and a 2 × 300 bp paired-end sequencing run was performed. The sequences generated passed through a filtering process to obtain the qualified reads. The total reads were merged, low-quality and chimera sequences were removed, and OTUs at a 97% similarity with the Greengenes database (v13.8) were clustered. We employed the QIAGEN CLC Microbial Genomics Module (v10.1.1) for further analysis.

### Processing and Statistical Analysis of Metataxonomic Data

The alpha diversity of taxonomic composition was measured using the Shannon diversity index, which evaluates the overall diversity of each group including the number of observed species (richness) and the evenness of the observed taxonomic composition. Beta diversity was measured using PCoA-Weighted UniFrac to determine the difference in microbial composition among groups. The OTU table was generated using the QIAGEN CLC Microbial Genomics Module combined with linear discriminant analysis effect size (LEfSe). LEfSe was performed using the Galaxy/Hutlab webtool to identify specific microbial markers among groups, with an 0.05 alpha value for the factorial Kruskal–Wallis test and pairwise Wilcoxon test and a linear discriminant analysis (LDA) score cutoff of 2.0. The results revealed that the functional pathways had a significantly different abundance at level 3 among the groups. Additionally, Spearman's correlation (calculated using the corrplot package v0.84) and principal component analysis (PCA; performed using the ade4 package v1.7-16) were applied through the use of R language (v4.0.2). A comparison of the groups was undertaken through two-tailed *t*-test analysis, and a *p* < 0.05 was considered statistically significant. We used GraphPad Prism 8 (GraphPad Software, San Diego, CA, USA) to identify taxonomic differences and generate relative abundance plots.

### Library Preparation and Sequencing on the Illumina Platform

For the *L. paracasei* strain GMNL-653, library preparation was conducted in accordance with the Illumina TruSeq Nano DNA Sample Preparation Kit protocol. First, 200 ng of genomic DNA was sonicated to approximately 550 bp. Fragmented DNA was processed to convert the overhangs into blunt ends, and the library size was selected using beads. After ends repair, a single “A” nucleotide was added to the 3′ ends of the blunt fragments, and the corresponding single “T” nucleotide on the adapter with index was ligated to the fragments. The library size was verified through electrophoresis, and the quantification was validated with the NanoPhotometer. All validated libraries were mixed, denatured, and diluted to the appropriate concentration for pair-end 250-cycle sequencing on MiSeq.

For the GMNL-855 and BCRC-16100 strains, 1 μg DNA per sample was used as the input material for the DNA sample preparations. Sequencing libraries were generated using NEBNext Ultra DNA Library Prep Kit for Illumina (New England Biolabs, Ipswich, MA, USA) following the manufacturer's recommendations, and index codes were added to attribute sequences to each sample. In brief, the DNA sample was fragmented through sonication to the size of 350 bp, and DNA fragments were then end-polished, A-tailed, and ligated using the full-length adaptor for Illumina sequencing with further PCR amplification. The PCR products were purified (AMPure XP); the libraries were then analyzed for size distribution by using an Agilent 2100 Bioanalyzer and quantified using real-time PCR. The clustering of the index-coded samples was performed on a cBot Cluster Generation System according to the manufacturer's instructions. After cluster generation, the library preparations were sequenced on an Illumina NovaSeq 6000 platform, and paired-end reads were generated.

### Sample Preparation and Sequencing on Oxford Nanopore Technologies GridION

DNA extraction was conducted in accordance with the EasyPure Genomic DNA Kit protocol (TransGen Biotech). Library preparation was performed following Oxford Nanopore Technologies (ONT) protocols in the form of native barcoding genomic DNA (with EXP-NBD103 and SQK-LSK108). Next, 400 ng of genomic DNA was fragmented with transposome randomly, with a barcode added at the same time. DNA was subsequently purified using AMPureXP beads and ligated using motor protein-complexed adapter to DNA ends. The library was quantified using the Qubit Fluorometer, and the prepared library was loaded onto a running buffer-primed flow cell for sequencing. Sequencing of the native genomic DNA was performed on a single R9.4/FLO-MIN106 flow cell on the GridION.

### Hybrid Assembly and Genome Annotation

Genomes were constructed through a hybrid assembly method of low-coverage ONT long-read (approximate mean of 6.2, 7.8, and 7.8 kbp for GMNL-653, GMNL-855, and BCRC-16100, respectively), sequencing for generating scaffolds and high-coverage Illumina short-read (550 bp) sequencing for determining the consensus sequence. The *de novo* hybrid genome assembly approach was applied using the MaSuRCA v3.3.1 assembler (20). Specifically, the MaSuRCA assembler first reduces high-coverage Illumina reads to long super reads and then aligns them with long ONT reads to generate even longer super reads (21). Illumina short reads and ONT long reads were input into the MaSuRCA assembler without any preprocessing, as recommended in the MaSuRCA documentation. In addition, the MaSuRCA uses a high coverage of error-prone long reads generated by ONT to construct consensus sequences for regions not captured by the short Illumina reads. Consequently, this approach combines the advantages of the accuracy of Illumina short reads and the coverage of ONT long reads. Furthermore, Benchmarking Universal Single-Copy Orthologs (BUSCO; v.4.0.0) (22) was used for genome completeness assessment through comparison with the lactobacillales_odb10 gene database, which contains 402 orthologs of 304 species (23). The comparison of each chromosome with the reference sequences was conducted using BLASTN and visualized with Easyfig (24).

The genome was annotated through a multipronged approach consisting of the use of Prokka software (25) and a combination of *ab initio* and evidence-driven gene prediction, including protein-coding regions and RNA genes (i.e., tRNA, rRNA). Then, functional classification of these annotated genes was carried out using eggNOG-mapper (26, 27) for annotation and classification in conjunction with the database of Clusters of Orthologous Groups (COGs) of proteins. The CRISPRFinder program was employed to search for CRISPR direct repeats and spacers (28). Mobile genetic elements such as conjugative plasmids, bacteriophages, and transposons are regarded as a determinant force of horizontal gene transfer. Plasmid identification and typing were conducted using PlasFlow (29), and the genomes were screened for prophages using the PHASTER tool (30); transposon sequences were predicted using RepeatMasker software. Additionally, genes related to the production of bacteriocins were predicted using BAGEL4 (31). Following annotation, the circular genome atlas was generated using the Circos visualization tool (32).

### 16S rRNA Phylogenetic Analysis

To evaluate the quality of genome assembly, we compared the genome sequence of our strains with that of 12 reported *Lacticaseibacillus* strains from four *L. paracasei* species. The publicly available 16S rRNA sequences of *Lacticaseibacillus* spp. (*L. acidophilus, L. amylovorus, L. casei, L. crispatus, L. delbrueckii, L. fermentum, L. gasseri, L. johnsonii, L. plantarum, L. reuteri, L. rhamnosus*, and *L. salivarius*) were retrieved from the National Center for Biotechnology Information nucleotide database (https://www.ncbi.nlm.nih.gov/nuccore/?term=Lactobacillus). Then, the phylogenetic tree was constructed using Molecular Evolutionary Genetics Analysis (MEGA X) software (33). In addition, the multiple sequence alignment program ClustalW was employed to reconstruct the molecular evolution tree using the maximum-likelihood estimation method, with genetic distances computed using the Kimura two-parameter model. Confidence values for the phylogenetic trees were estimated through bootstrap analyses with 1,000 replicates.

### Whole-Genome Average Nucleotide Identity Analysis

Although the 16S rRNA sequence-based division into higher taxa is the most widely used classification system for prokaryotes, the sequence of 16S rRNA genes is too conservative to distinguish between closely related species. Hence, to measure the nucleotide-level genomic similarity between our strain and other related *Lactobacillus* genomes, the OrthoANI (34), regarded as the gold standard for prokaryotes at the genomic level, was calculated using the BLASTN program based on the modified algorithm of average nucleotide identity (ANI) (35).

### Determination of Virulence Factors and Antimicrobial Resistance Properties

After genome annotation, each homolog of the *L. paracasei* genes was searched in the BLAST database against the Integrated Microbial Genomes and Microbiomes database v.5.0 (36), Virulence Factor Database (VFDB) (37), and Comprehensive Antibiotic Resistance Database (CARD) (38) to identify candidate virulence genes and antibiotic-resistant genes using genotypic methods. The protein-coding gene was submitted into the Resistance Gene Identifier in the CARD for identification of antimicrobial resistance genes under the default settings (perfect/strict option). The BLAST hit cutoff values, and an *E* <1E-30 were used to identify the possible virulence genes and antibiotic-resistant genes for further investigation of mobile genetic elements such as bacteriophages, plasmids, naked DNA, or horizontal transposons. In addition, the BlastKOALA (39) search tool v.2.2 in the Kyoto Encyclopedia of Genes and Genomes (KEGG) database (https://www.kegg.jp/) was used for the investigation of undesirable genes.

## Results

### Heat-Killed *L. paracasei* (GMNL-653) Reduced Levels of IL-6 and NO in LPS-Stimulated Mouse RAW 264.7 Macrophages

**Figure 1:**
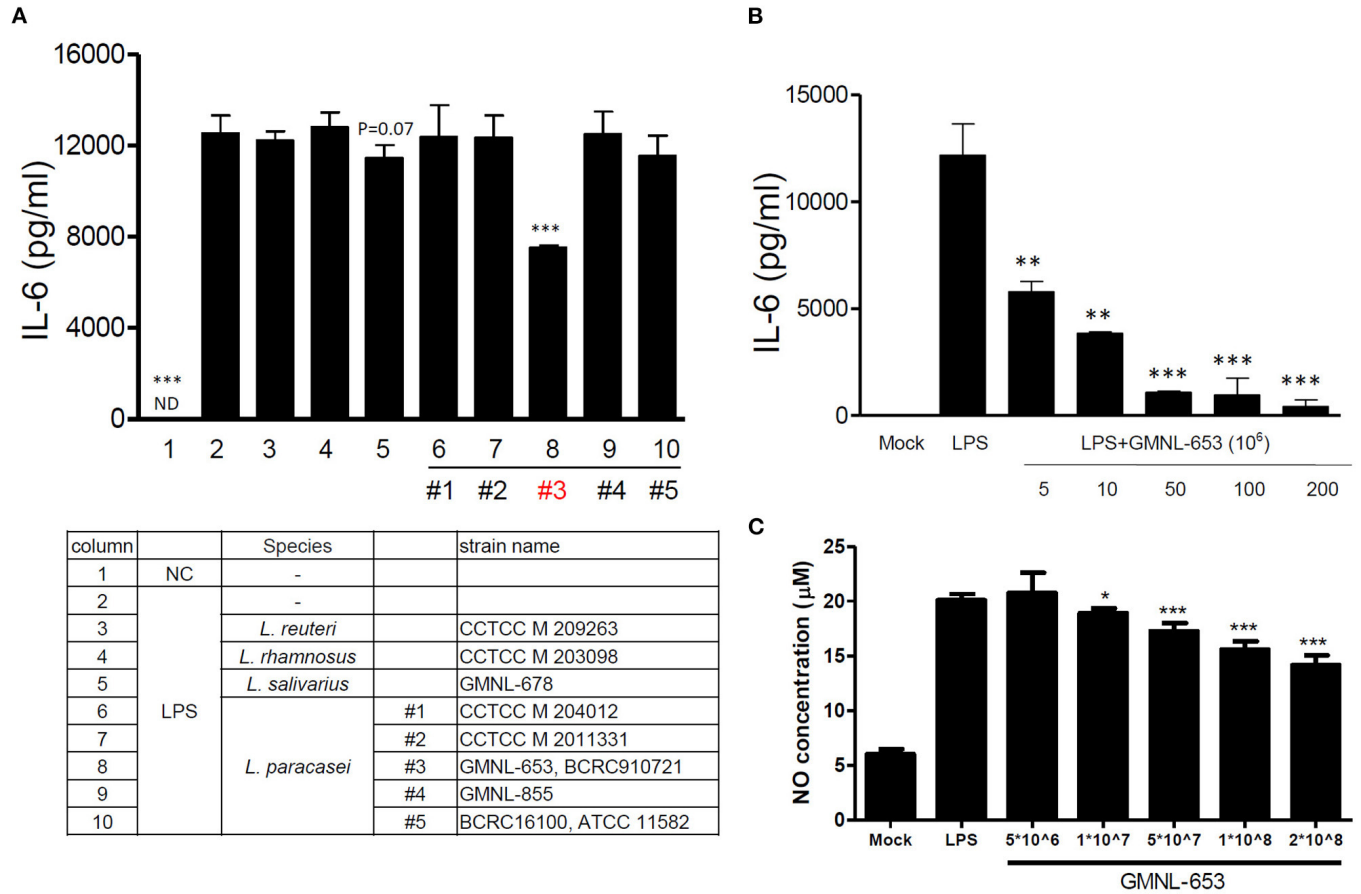
Heat-killed probiotics reduced IL-6 and NO levels through LPS stimulation in mouse RAW 264.7 macrophages. An appropriate number of cells were seeded in a 24-well plate. Probiotics (*L. reuteri, L. rhamnosus, L. salivarius*, and five strains of *L. paracasei*) were cultured in MRS broth for 20 h, with the bacterial concentration adjusted through centrifugation. These bacteria were heat killed (90°C for 30 min) and then added to cells with or without LPS stimulation. After 20 h, the culture supernatant was collected for measurement of the IL-6 production **(A,B)** and NO **(C)**. *P < 0.05, **P < 0.01, and ***P < 0.001 compared with the group treated with LPS alone.

The inflammation and bone cells are highly related. We used the macrophage *in vitro* model to screen a variety of strains of heat-killed probiotics with anti-inflammatory activity, namely *L. reuteri, L. rhamnosus, L. salivarius*, and *L. paracasei*. These bacteria were inactivated at 90 °C for 30 min and then added to mouse RAW264.7 macrophages with or without LPS stimulation. Our results revealed that only *L. salivarius* GMNL-678 *and L. paracasei* GMNL-653 (Figure 1A, column 5 and 8) reduced LPS-induced IL-6 production in the macrophages. The other *Lactobacilli* had no inhibitory effect on the production of IL-6 and NO in LPS-stimulated macrophages (Figure 1A). GMNL-653 inhibited IL-6 and NO production under LPS stimulation in a dose-dependent manner (Figures 1B,C).

### Heat-Killed GMNL-653 Increased Calcium Mineralization in Human Osteosarcoma MG63 Cells in Osteoinduction Medium

**Figure 2:**
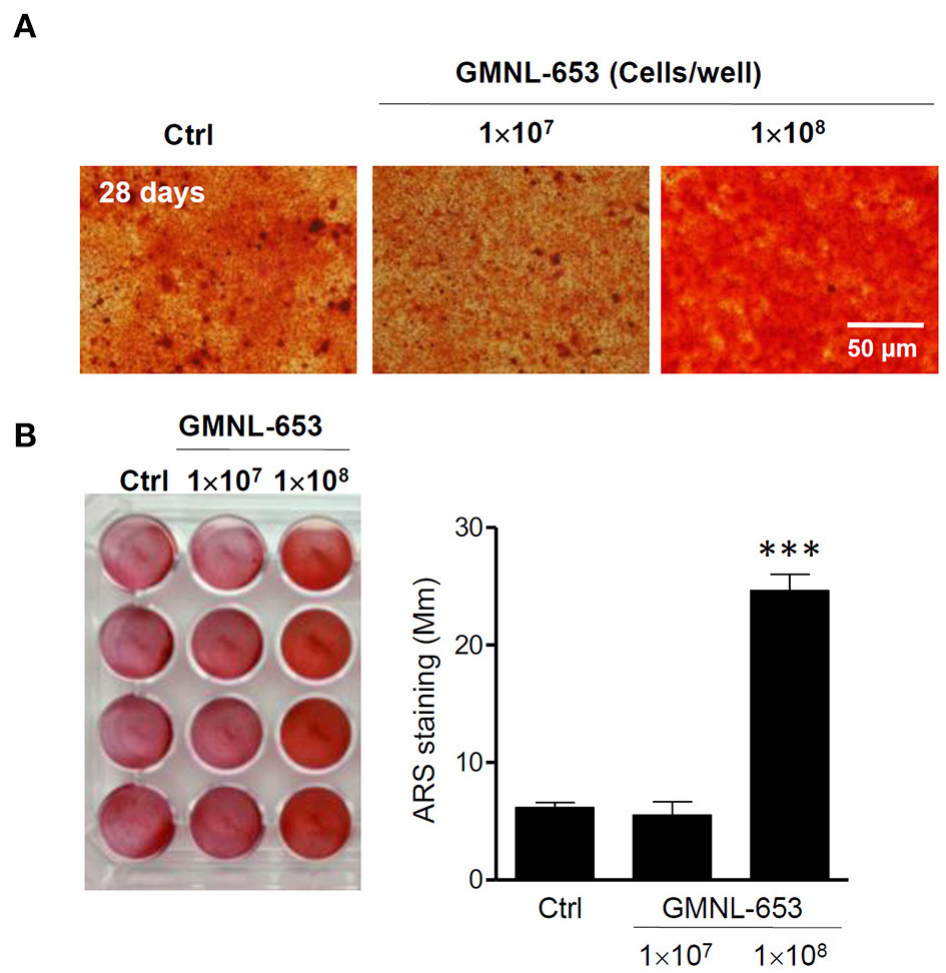
Heat-killed *L. paracasei* (GMNL-653) increased calcium mineralization in human osteosarcoma MG63 cells mineralizing in culture. **(A)** Human osteosarcoma MG63 cells are incubated with heat-killed GMNL-653 in osteoinduction medium for 28 days. Following fixation, the cells are stained with Alizarin Red S to measure calcium deposits. **(B)** Quantification of Alizarin Red S stain by the absorbance (405 nm) of the red extracts according to the manufacturer's instructions. ***P < 0.001 compared with the control group.

Bone is a mineralized tissue that continuously undergoes remodeling, with homeostasis achieved through the balance of osteoblasts and osteoclasts (40). First, we employed ARS staining to evaluate calcium deposits using *in vitro* culture (41), demonstrating that a high dose (1 × 10^8^/mL) of GMNL-653 markedly increased calcium deposits in the MG63 cells (Figure 2). Furthermore, we investigated whether heat-killed GMNL-653 triggered the differentiation of macrophages into osteoclasts. Acid phosphatase-positive multinucleated (>3 nuclei) cells were counted as osteoclasts, revealing that GMNL-653 did not influence osteoclast differentiation under RANKL stimulation *in vitro* (Supplementary Figure 1).

### GMNL-653 Protects Mice From Ovariectomy-Induced Bone Loss

**Figure 3:**
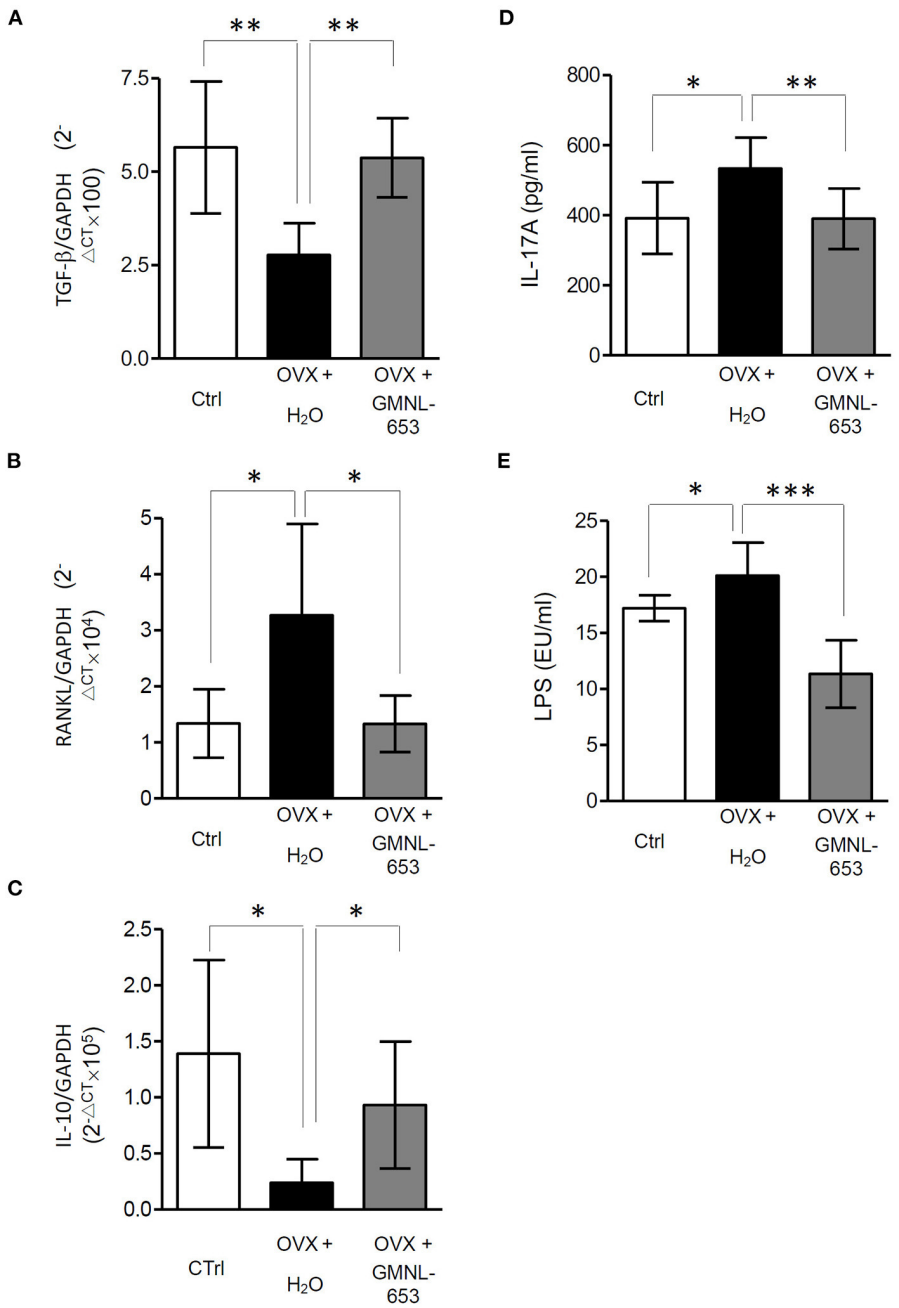
**(A–C)** The mRNA levels of *TGF-*α*, RANKL*, and *IL-10* in the tibia. **(D,E)** The IL-17A and LPS levels in the sera of ovariectomized (OVX) mice treated with either the vehicle (H_2_O) or heat-killed *L. paracasei* (GMNL-653). qRT-PCR analysis of the expression of genes known to promote bone formation, *TGF-*β and *IL-10* **(A,C)**, and bone resorption, *RANKL*, in the tibia of OVX mice. The levels of IL-17A and LPS in the sera are measured through ELISA. *P < 0.05, **P < 0.01, and ***P < 0.001 compared with the OVX+H_2_O group.

To determine the effect of heat-killed probiotic GMNL-653 treatment on OVX-induced bone loss, 8-week-old ICR mice were treated with a vehicle (H_2_O), GMNL-653, and GMNL-678 once a day following OVX or sham surgery for 28 days. Alendronate treatment acted as a positive control to treat and prevent osteoporosis in OVX mice (Table l). We performed high resolution micro-computed tomography for evaluation right tibia bone morphology and architecture with treatment GMNL-653 in OVX mice. The result showed that GMNL-653 enhances bone microarchitecture in OVX mice (Supplementary Figure 2). In the vehicle-treated mice, OVX decreased BV/TV and BMD, whereas the GMNL-653-treated mice exhibited increased BV/TV and BMN. The effects of GMNL-678 were not as obvious as those of GMNL-653. Decreased mRNA levels of *TGF-*β and *IL-10* and increased *RANKL* in the tibia were observed in the OVX mice treated with H_2_O (Figures 3A–C). However, GMNL-653 restored the *TGF-*β and *IL-10* levels and decreased the *RANKL* mRNA levels in the OVX mice. We also detect mRNA levels of *bone morphogenetic proteins 2 (BMP2)* and *tartrate-resistant acid phosphatase 5b (TRAP-5)* in OVX mice (Supplementary Figure 3). BMP-2 plays a critical role in osteoblast differentiation (42). TRAP-5 has been used as a reliable biomarker of bone resorption and osteoclast number (43). There has been an increasing trend in mRNA levels of *BMP-2* in GMNL-653 treated OVX mice. The GMNL-653 decreased *TRAP-5* levels in OVX mice. In the OVX mice, IL-17A, and LPS levels in the sera also increased, though GMNL-653 decreased these levels (Figures 3D,E). These data indicated that GMNL-653 protects mice from ovariectomy-induced bone loss and enhances gut barrier integrity.

**Table 1:** Heat-killed *L. paracasei* (GMNL-653) protect mice from ovariectomy-induced bone loss.

**Control**	**OVX +** **H_**2**_O**	**OVX +** **GMNL-653**	**OVX +** **GMNL-678**	**OVX +** **alendronate**
**BV/TV (%)**				
42.12 ± 2.4**	30.9 ± 1.1	36.80 ± 1.6**	32.38 ± 0.8*	34.88 ± 0.9**
**BMD (g/cm** ^**3**^ **)**				
0.502 ± 0.04**	0.344 ± 0.04	0.444 ± 0.043**	0.38 ± 0.027*	0.426 ± 0.02*

* *P < 0.05 and*

** *P < 0.01 compared with the OVX+H_2_O group*.

### GMNL-653 Modulated Gut Microbiota Composition in OVX Mice

**Figure 4:**
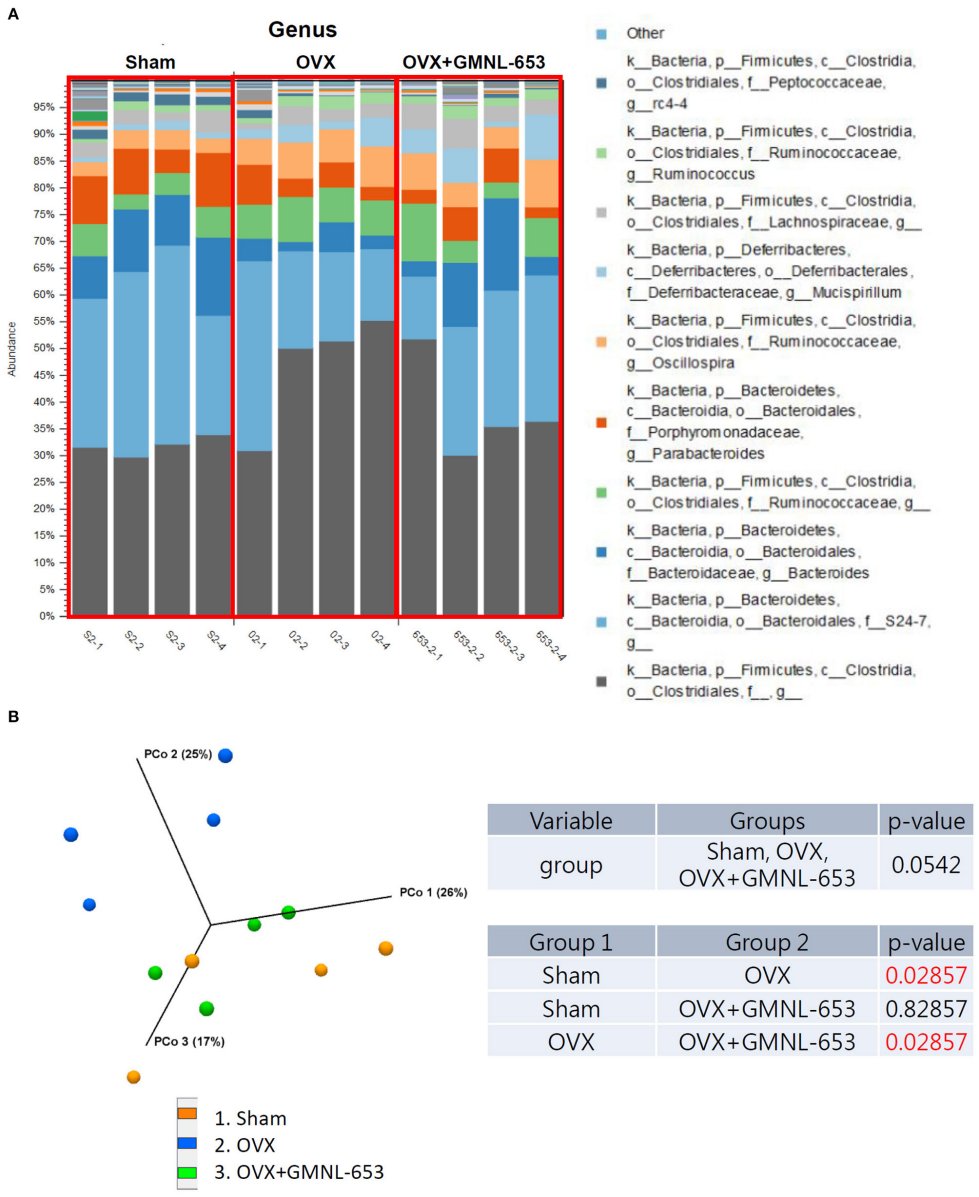
Relative abundance and diversity of stool microbiota in the sham and OVX mice treated with either the vehicle (H_2_O) or heat-killed GMNL-653. **(A)** Each column in the stacked histogram represents one sample; the differently colored bars are proportional to the percentage of relative bacterial abundance of each taxon within the sample (summing up to 100%). **(B)** Principal coordinate analysis of the beta diversity among the three groups.

Because gut barrier integrity may have been affected in the OVX mice, we further analyzed the gut microbiota compositions. Relative abundance (Figure 4A) and diversity (Figure 4B) of the fecal microbiota in the sham and OVX mice treated with either the vehicle (H_2_O) or GMNL-653 are depicted; at the genus level, the dominant gut microbiota were *rc4-4, Ruminococcus, Lachnospiracear, Mucispirillum, Oscillospira, Parabacteroides, Ruminococcaceae, Bacteroides, S24-7*, and *Clostridiales* (Figure 4A). The results of the PCoA plot of beta diversity (Figure 4B) indicated that the microbial community, compared with the sham, OVX, and GMNL-653-treated OVX mice groups, exhibited a significantly distinct cluster that was separate from the other groups.

**Figure 5:**
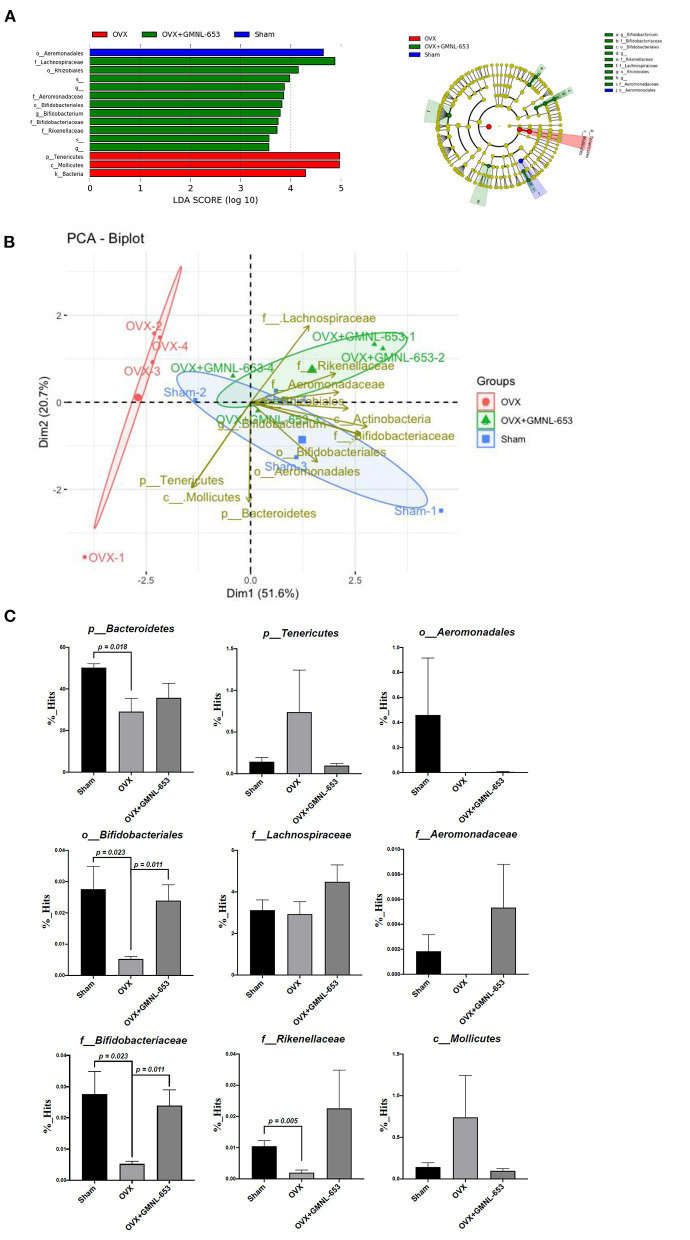
Taxonomic differences and relative abundance of fecal microbiota in the sham and OVX mice treated with either the vehicle (H_2_O) or heat-killed GMNL-653. **(A)** Linear discriminant analysis and cladogram revealed the phylogenetic distribution of fecal microbiota associated with the sham, OVX, and OVX + GMNL-653 groups. The biplot of the principal component analysis **(B)** and relative abundances **(C)** of bacterial communities in the sham, OVX, and OVX+GMNL-653 groups.

The differential microbiota from the three groups were identified through LEfSe analysis (Figure 5A), which demonstrated that *Tenericutes* (phylum) and *Mollicutes* (class) were enriched in the OVX group and *Lachnospiraceae* (family), *Rhizobiales* (order), *Aeromonadaceae* (family), *Bifidobacteriales* (order), *Bifidobacterium* (genus), *Bifidobacteriaceae* (family), and *Rikenellaceae* (family) were enriched in the OVX+GMNL-653 group. A PCA biplot was generated to investigate the microbial composition differences between the sham, OXV, and OVX + GMNL-653 groups (Figure 5B). This biplot revealed that dimension 1 and dimension 2 explained 51.6 and 20.7% of the variation in gut microbiota composition, respectively. Obvious intergroup distances tended to form distinct clusters between the OVX group and the other two groups, indicating dissimilar gut microbiota. These results suggested that dysbiosis occurred in the OVX mice, which was then modulated through GMNL-653 intervention, leading to a similar configuration to the sham control group. The relative abundance of gut bacteria indicated that the enrichment of *Tenericutes* (phylum) and *Mollicutes* (class) in the OVX mice could be reduced through GMNL-653 supplementation. Likewise, the decreased *Bifidobacteriales* (order), *Bifidobacteriaceae* (family), and *Rikenellaceae* (family) abundance in the OVX mice was enriched by GMNL-653 (Figure 5C).

### GMNL-653 Intervention Influenced the Relative Abundance of the PICRUSt Functional Prediction of Colonic Microbiota in OVX Mice

**Figure 6:**
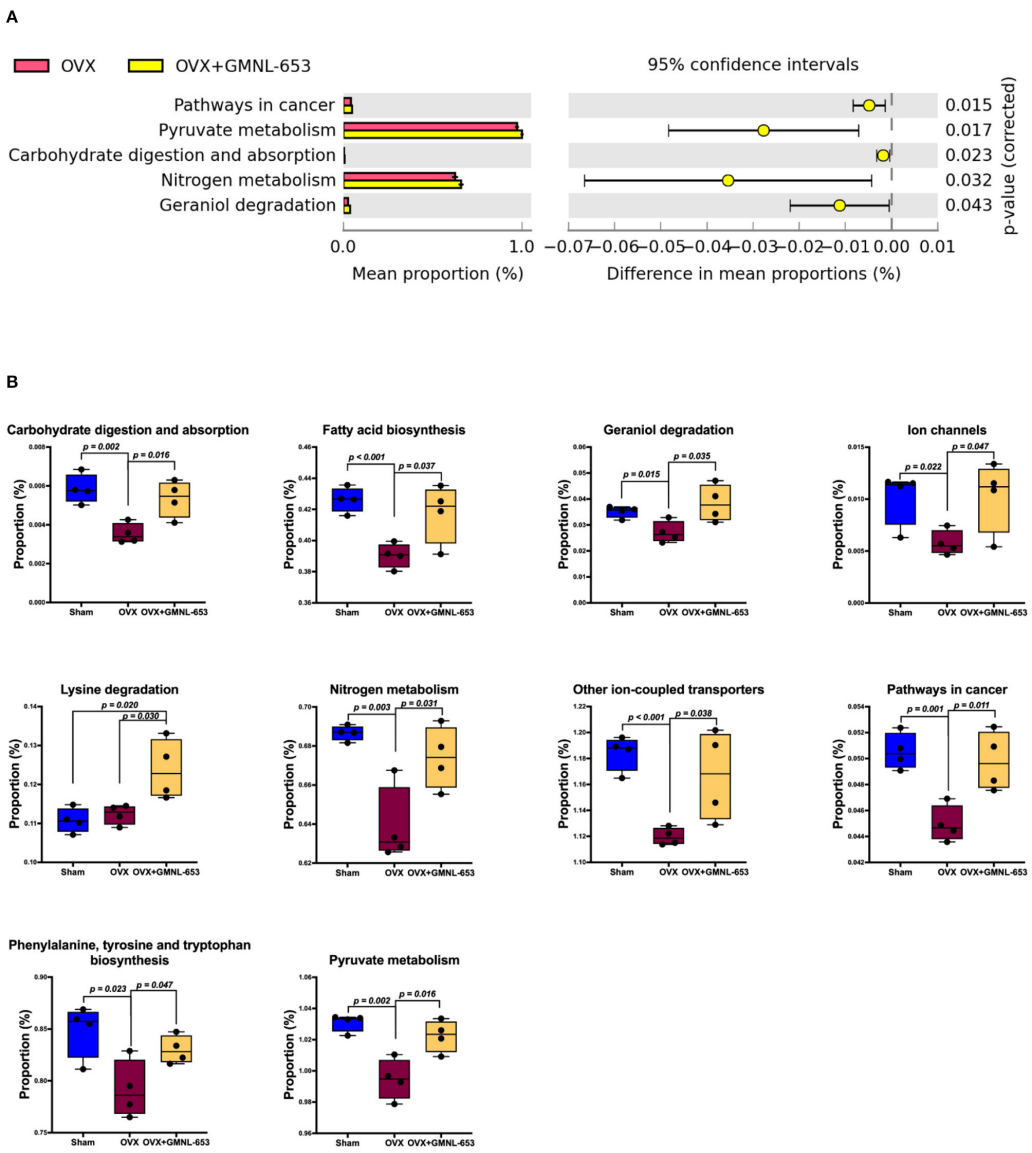
Predicted function of the involved gut microbial using the phylogenetic investigation of communities by the reconstruction of unobserved states 2 (PICRUSt2) in the sham and OVX mice treated with either the vehicle (H_2_O) or heat-killed GMNL-653. **(A)** Pairwise comparison showed that five function pathways were significantly increased in mice treated with GMNL-653 vs. OVX mice. Among them, 4 of 5 function pathways were included in the metabolism of pyruvate, carbohydrate, nitrogen, and geraniol. **(B)** There were nine function pathways overlapped between OVX vs. GMNL-653 and Sham vs. OVX and were significantly decreased in the OVX mice as compared to the Sham group, but further increased upon GMNL-653 treatment. It is noted that only Lysine degradation pathway was overlapping between OVX vs. GMNL-653 and Sham vs. GMNL-653 and was significantly increased in the OVX mice as compared to the Sham group, but was further increased when treated with GMNL-653. *p* < 0.05 is considered as statistically significant.

To investigate the effect of GMNL-653 on the functional profiles of microbiome communities in the OVX mice, we used PICRUSt to predict metagenomes based on 16S rRNA marker gene sequences and the KEGG database. The results revealed that the relative abundance of several metabolic pathways associated with pathways of cancer, pyruvate metabolism, carbohydrate digestion and absorption, nitrogen metabolism, and geraniol degradation in the colon of OVX mice were altered through GMNL-653 treatment (Figure 6A). The relative abundance of the functional pathways in carbohydrate digestion and absorption, fatty acid biosynthesis, ion channels, nitrogen metabolism, and other ion-coupled transporters as well as the pathways of cancer, phenylalanine, tyrosine, and tryptophan biosynthesis, and pyruvate metabolism were downregulated in the OXV group compared with those in the sham group; they were, however, upregulated through GMNL-653 treatment (Figure 6B).

### Correlation Between Gut Microbiota Composition and Sera Concentrations of IL-17 and LPS in OVX Mice

**Figure 7:**
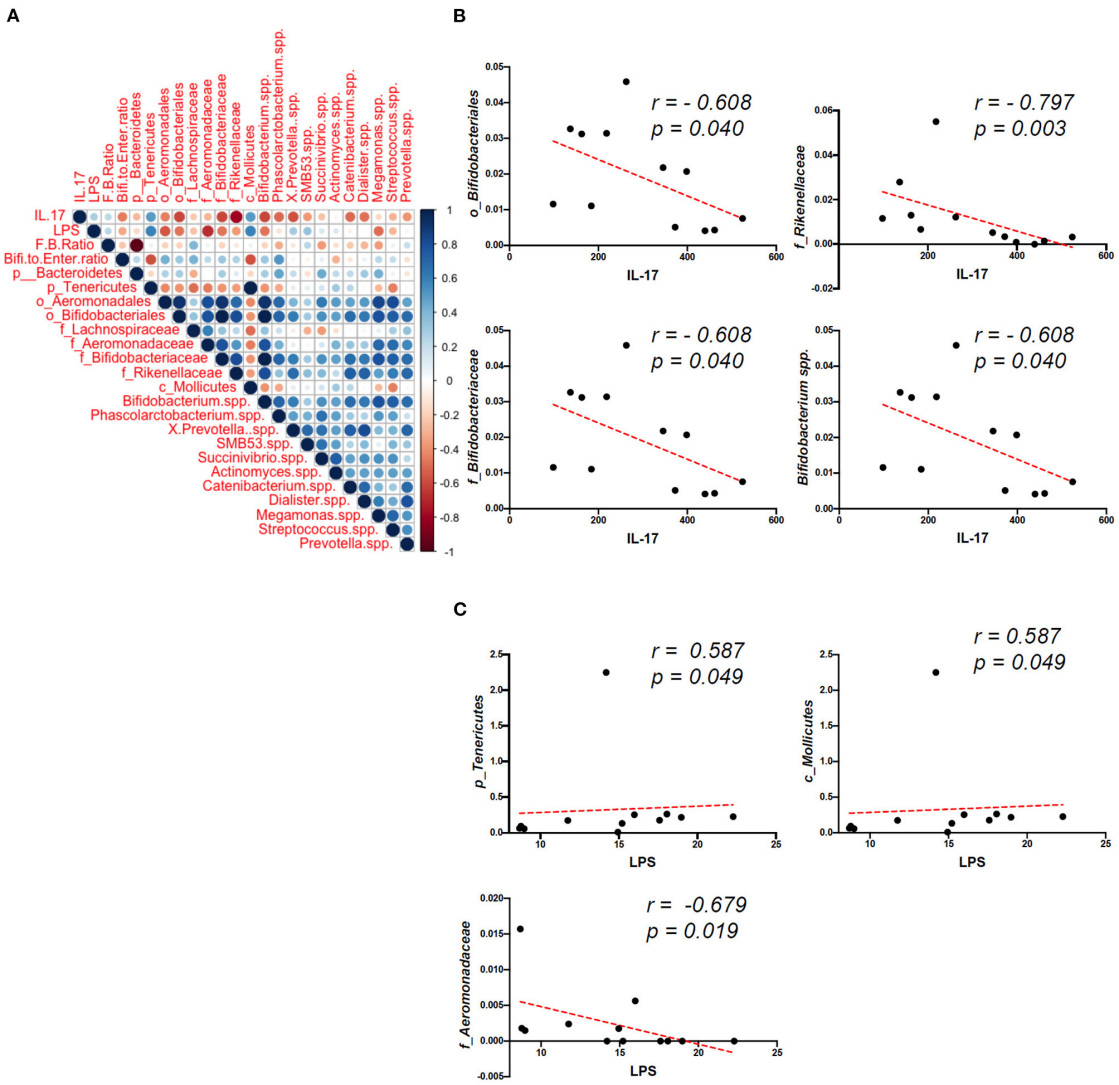
Correlations between the gut microbiota composition and plasma concentrations of IL-17 and LPS in the sham and OVX mice treated with either the vehicle (H_2_O) or heat-killed GMNL-653. **(A)** Spearman's correlation coefficient strength of the phylum counts from the OVX mice. Correlations with *P* < 0.05 are indicated, and the strength of the coefficient is represented by the change in color intensity. **(B)** The enriched *Bifidobacteriales* (order), *Rikenellaceae* (family), *Bifidobacteriaceae* (family), and *Bifidobacterium* spp. in the OVX + GMNL653 group exhibited a significant negative correlation with sera IL-17. **(C)** The enriched *Tenericutes* (phylum) and *Mollicutes* (class) in the OVX group were positively correlated with sera LPS. The enriched *Aeromonadacae* (family) in the OVX + GMNL-653 group exhibited a negative correlation with sera LPS.

Our results indicated that sera IL-17 and LPS increased in the OVA mice, implying that gut microbiota composition may be associated with immune responses to osteoporosis. Because GMNL-653 intervention could reverse the dysbiosis of gut microbiota, we further investigated the associations between gut microbiota composition and IL-17 and LPS levels in the OVX mice. We then assessed which specific species were enriched or depleted in the OVA mice and whether this correlated with the cytokine and LPS concentrations. Using Spearman's correlation coefficient, we also investigated the correlation strength of the phylum counted in the OVX mice (Figure 7A), with the results demonstrating that the enriched *Bifidobacteriales* (order), *Rikenellaceae* (family), *Bifidobacteriaceae* (family), and *Bifidobacterium* spp. in the OVX + GMNL653 group had significantly negative correlations with sera IL-17. Moreover, the enriched *Tenericutes* (phylum) and *Mollicutes* (class) in the OVX group exhibited positive correlations with sera LPS, whereas the enriched *Aeromonadacae* (family) in the OVX + GMNL-653 group was negatively correlated with sera LPS (Figure 7B).

### General Genome Features and Comparative Genome Analysis

Whole-genomic DNA–DNA hybridization (DDH) is the gold standard for bacterial species delineation (44). NGS-based genome sequencing has also been widely applied to the taxonomy of microorganisms and was successfully used to distinguish between *L. casei* and *L*. *paracasei* strains (45). However, these technologies are time intensive, costly, and difficult to use routinely in laboratories. Comparison of the DNA sequences of protein-encoding genes is an alternative approach to the analysis of whole-genome relatedness and has become increasingly critical in the use of these sequences as molecular targets for microbial species identification. We identified and verified the distinct nature of the three strains of *L. paracasei* (GMNL-653, GMNL-855, and BCRC-16100) by using SEM, RAPD analysis, and carbohydrate fermentation features (Supplementary Figure 4). Furthermore, we performed genome sequencing and characterization of the strains to identify genes potentially involved in probiotic activity.

**Figure 8:**
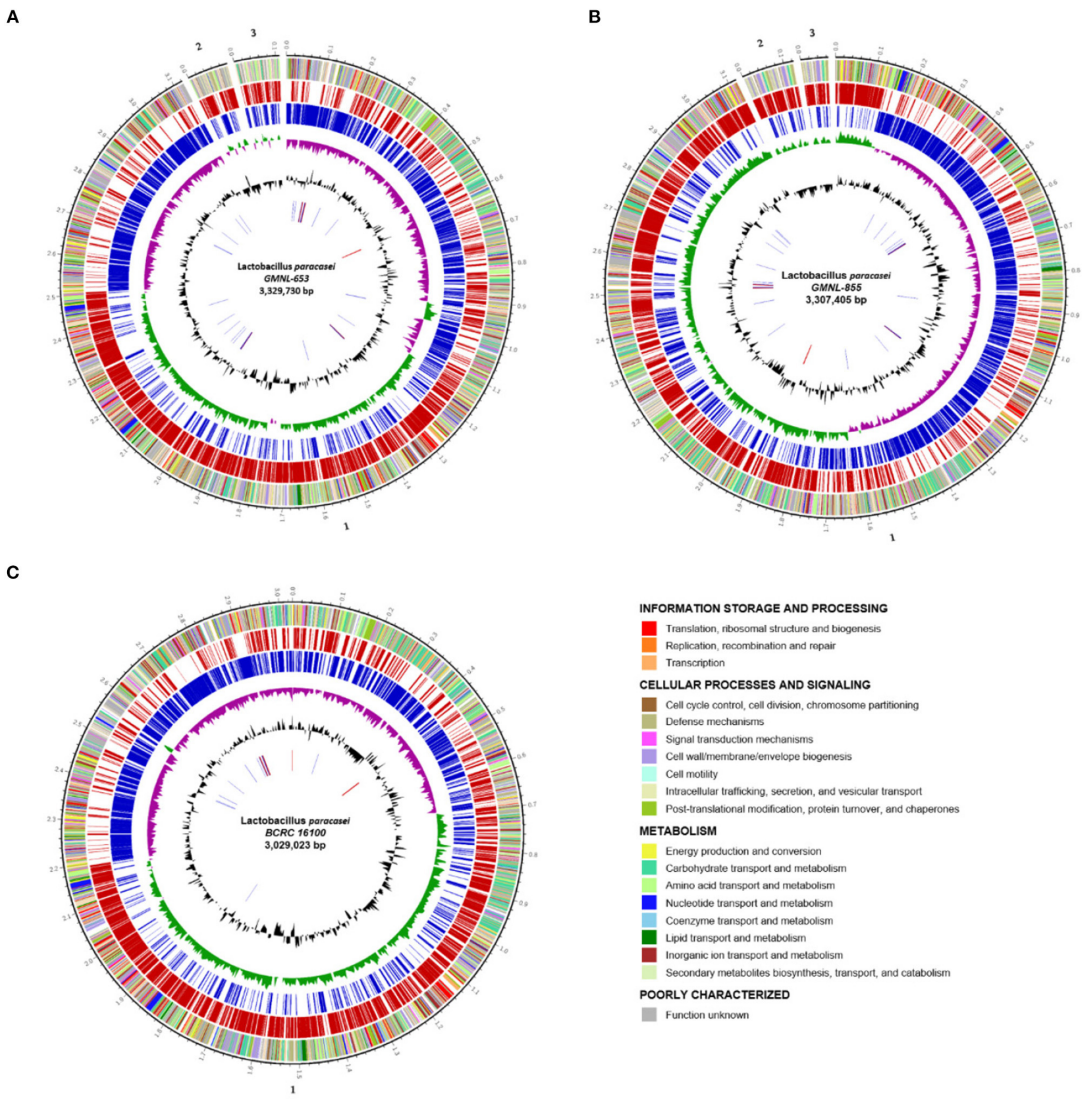
Circular genome graph **(A–C)** of the *L. paracasei* strain. **(A)** The GMNL-653, **(B)** GMNL-855, and **(C)** BCRC-16100 genome. The outer to inner circles depict the (1) DNA coordinates; (2) function-based color-coded mapping of the genes, colored according to the COG category whose code and meaning is provided in the table below the plot; (3, 4) coding sequences predicted on the forward (red) and reverse (blue) strands, respectively; (5) GC skew (G – C/G + C), calculated for a window of 4,000 bp and shifted by 1,000 bp over the genome sequence, with the above- and below-average regions indicated in green and purple; (6) GC content (black); and the (7) RNA genes (tRNA, blue; rRNA, red; other RNAs, black).

Three *L. paracasei* strains (GMNL-653, GMNL-855, and BCRC-16100) were characterized at the genotypic and phenotypic level (Figure 8). High-molecular-weight DNA from three isolates of the *L. paracasei* strain was sequenced using ONT long reads and Illumina short reads for the hybrid assembly of their genomes using the MaSuRCA assembler v3.3.1 (20). As detailed in Table 2, the assembly of the GMNL-653 draft genome sequence consists of three contigs amounting to 3,329,730 bp. The first contigs (3,121,847 bp) were regarded as a circular chromosome predicted using PlasFlow (29), with a GC content of 46.19%. Prokka (25) software was used to annotate 3,375 protein-coding genes and identify 60 tRNA genes and five rRNA operons. The genetic organization of GMNL-653 was illustrated as a genome atlas constructed with Circos software (Figure 8A) (32). For completeness assessment for genomics data quality control, BUSCO analysis was employed, revealing that 400 of the 402 reference genes were complete (99.5%, 391 single-copy and 9 duplicated), 2 (0.5%) were fragmented, and none were missing in the draft assembly; thus, the assembly represents most of the genomic content (Supplementary Figure 5). One separate CRISPR loci (Table 2, Supplementary Table 1) was discovered in the genome of GMNL-653 by using the CRISPRFinder (28). Additionally, eight prophage regions were identified, of which four regions were intact, two were incomplete, and two were questionable (Supplementary Table 2); a type IIa bacteriocin operon (Supplementary Table 3) was also identified in GMNL-653.

**Table 2:** General features of *L. paracasei* genomes.

	**GMNL-653**	**GMNL-855**	**BCRC 16100**
Size (bp)	3,329,730	3,307,405	3,029,023
G + C content (%)	46.19	46.19	46.4
Total genes	3,375	3,328	2,902
Coding content (%)	88.82	86.27	85.88
Gene average length (bp)	852	857	896
Genes assigned to COGs	2,627 (77.8%)	2,669 (80.2%)	2,387 (82.2%)
Chromosome	1	1	1
rRNA operons	5	5	5
tRNA	60	60	62
Plasmids	0	0	0
Transposases	16	104	13
CRISPR loci	1	1	2
Prophage-Like clusters	4	5	0
Bacteriocin	3	3	2

The complete genome sequence of GMNL-855 and BCRC-16100 comprised a circular chromosome (3,118,787 and 3,029,023 in contigs 1, respectively), with average GC values of 46.4 and 46.19%, respectively. Figures 8B,C illustrates the orthologous genes shared among strains, presenting the position and color-coded function of each specific gene. Through BUSCO analysis, we determined that 99.5% of reference genes were complete in both strains, 2 (0.5%) were fragmented, and none were missing (Supplementary Figure 5). In total, over 99% of complete BUSCOs were present in the three *L. paracasei* strains, indicating high-quality assemblies. Of the 3,328 and 2,902 genes that were predicted in GMNL-855 and BCRC-16100, respectively, 86.27 and 85.88% were protein-coding genes, respectively. Gene prediction demonstrated the presence of the following putative genes: three CRISPR/CRISPR-associated (cas) gene loci (type I and IIA; Supplementary Table 1), five intact prophage-like regions (Supplementary Table 2), and several type IIb bacteriocin operons (Supplementary Table 3).

### Genotypic Identification of the *L. paracasei* Strain

The beneficial health attributes and safety concerns of probiotic cultures are closely related; thus, categorical identification of the probiotic strain is paramount (46). A maximum-likelihood tree of four *L. paracasei* genomes and 12 reported *Lactobacillus* strains (*L. acidophilus, L. amylovorus, L. casei, L. crispatus, L. delbrueckii, L. fermentum, L. gasseri, L. johnsonii, L. plantarum, L. reuteri, L. rhamnosus*, and *L. salivarius*) was created based on 16S rRNA gene sequences. Genome-wide phylogenetic analysis in combination with alignment fraction analysis indicated that GMNL-653, GMNL-855, BCRC-16100, and *L. casei* spp. were grouped together (Supplementary Figure 6). Another work documented an approximately 90% sequence identity among *L. casei, L. paracasei*, and *L. rhamnosus* (47). Furthermore, to measure genomic similarity at the nucleotide level between GMNL-653 and other *Lactobacillus* genomes, the average nucleotide identity (ANI) was calculated using an improved algorithm (34). The ANI values were calculated and revealed that three *L. paracasei* strains were most closely related to *L. paracasei LC2W* in terms of their nucleotide sequences (ANI > 98%; Supplementary Figure 7). The values between the two strains were extracted from the junction point of the diagonals departing from each strain. The critical ANI value aligned to other species was between 64.94 and 79.55%. A BLAST comparison of GMNL-653 against the GMNL-855 and BCRC-16100 strains was performed using Easyfig (Supplementary Figure 8). Genome-wide comparison indicated a high degree of synteny among the characterized *L. paracasei* GMNL-653, GMNL-855, and BCRC-16100 strains, with only a few regions disrupted throughout the chromosome. Therefore, the whole-genome data verified that the GMNL-653 strain belongs to the species *L. paracasei*. The functional classification of Clusters of Orthologous Groups (COGs) predicted using eggNOG-mapper (26, 27) revealed that 2,627 (77.8%) putative coding sequences were homologous to known gene families. The distribution of genes into COG functional categories is summarized in Table 3. Functional analysis of GMNL-653 genes revealed that, in addition to hypothetical proteins, a relative abundance of the gene was involved in the basic mechanisms of DNA replication and recombination, carbohydrate metabolism, and cell wall/membrane/envelope biogenesis. Additionally, in the putative regions of genomic coding, 2,669 (80.2%) and 2,387 (82.2%) genes were attributed to a COG family (Table 3). A core- and pan-genome analysis indicated that 72, 40, and 34 genes were specific to GMNL-653, GMNL-855, and BCRC-16100, respectively (Supplementary Table 4).

**Table 3:** COG functional categories of *L. paracasei* strain.

**COG class**	**Description**	**GMNL-653**	**GMNL-855**	**BCRC 16100**
J	Translation, ribosomal structure, and biogenesis	107 (4.1%)	157 (5.9%)	149 (6.2%)
L	Replication, recombination, and repair	304 (11.6%)	258 (9.7%)	135 (5.7%)
K	Transcription	209 (8.0%)	212 (8.0%)	201 (8.4%)
D	Cell cycle control, cell division, chromosome partitioning	29 (1.1%)	28 (1.1%)	25 (1.0%)
V	Defense mechanisms	104 (4.0%)	103 (3.9%)	81 (3.4%)
T	Signal transduction mechanisms	60 (2.3%)	62 (2.3%)	64 (2.7%)
M	Cell wall/membrane/envelope biogenesis	129 (4.9%)	136 (5.1%)	138 (5.8%)
N	Cell motility	2 (0.1%)	1 (0.1%)	1 (0.1%)
U	Intracellular trafficking, secretion, and vesicular transport	20 (0.8%)	24 (0.9%)	16 (0.7%)
O	Post-translational modification, protein turnover, and chaperones	71 (2.7%)	65 (2.4%)	61 (2.6%)
C	Energy production and conversion	107 (4.1%)	113 (4.2%)	110 (4.6%)
G	Carbohydrate transport and metabolism	270 (10.3%)	314 (11.8%)	307 (12.9%)
E	Amino acid transport and metabolism	207 (7.9%)	203 (7.6%)	196 (8.2%)
F	Nucleotide transport and metabolism	88 (3.3%)	88 (3.3%)	87 (3.6%)
H	Coenzyme transport and metabolism	47 (1.8%)	46 (1.7%)	47 (2.0%)
I	Lipid transport and metabolism	55 (2.1%)	54 (2.0%)	52 (2.2%)
P	Inorganic ion transport and metabolism	113 (4.3%)	113 (4.2%)	114 (4.8%)
Q	Secondary metabolites biosynthesis, transport, and catabolism	18 (0.7%)	13 (0.5%)	16 (0.7%)
S	Function unknown	687 (26.2%)	676 (25.4%)	587 (24.6%)

**Figure 9:**
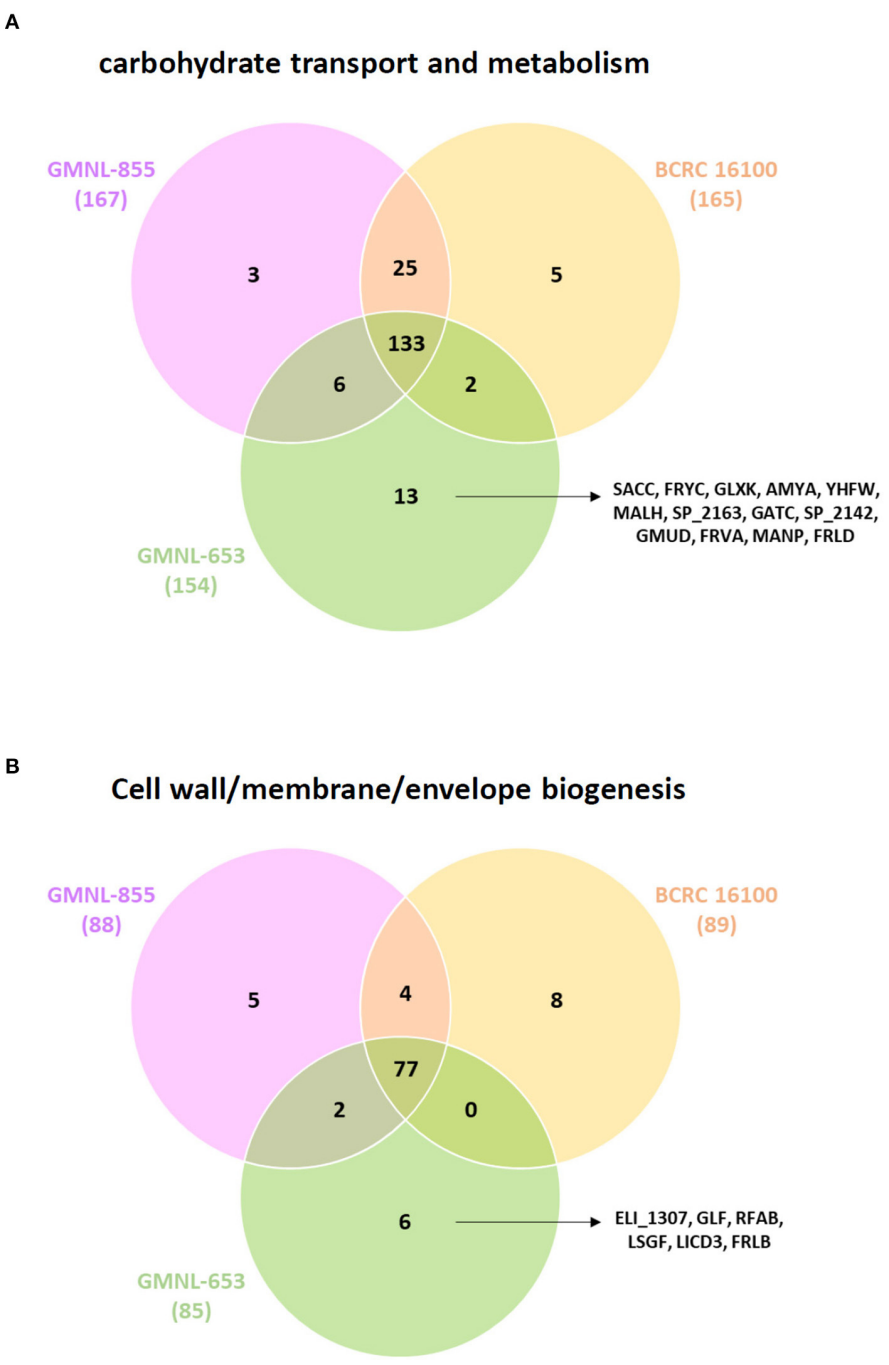
Comparison of the genes related to **(A)** carbohydrate transport and metabolism and **(B)** cell wall/membrane/envelope biogenesis among the three *L. paracasei* strains.

Cell wall components are key mediators influence on the interactions between host and associated microbiota. *Lacticaseibacillus* not only utilize and metabolize a variety of carbohydrates but also synthesized extensive exopolysaccharides which may affect other commensal bacteria resulting in health benefits in host. Therefore, genome sequencing of many *Lactobacillus* strains have revealed many genes involved in the metabolism of carbohydrate. Based on our results, we proposed that the bacterial cell wall components or carbohydrate metabolism exclusively expressing in GMNL-653 may be involved in the antiosteoporotic activity. We compared genes related to carbohydrate transport and metabolism (Figure 9A) and cell wall/membrane/envelope biogenesis (Figure 9B) among the three *L. paracasei* strains. Thirteen genes were annotated to carbohydrate transport and metabolism, which are sucrose-6-phosphate hydrolase, phosphotransferase (PTS) system, glycerate kinase, sucrose phosphorylase, phosphotransfer between the C1 and C5 carbon atoms of pentose, glycoside hydrolase family 4, repressor, ORF, kinase (ROK) family, glycosyl hydrolase family 1, and sugar kinase (Table 4). Six genes were related to cell wall/membrane/envelope biogenesis, which are phage tail tape measure protein, udp-galactopyranose mutase, glycosyl transferase, licD family, and catalyzes the conversion of a range of fructosamine 6-phosphates to glucose 6phosphate, and a free amino acid (Table 4). According to the KEGG BRITE database, these thirteen genes annotated to carbohydrate transport and metabolism have functional hierarchy classification of starch and sucrose metabolism, fructose-specific II-like component, biosynthesis of secondary metabolism, pentose phosphate pathway/purine metabolism, mitochondrial DNA transcription and translation factors, fructose like PTS system EIIA component, and fructoselysine 6-kinase. Six genes related to cell wall/membrane/envelope biogenesis have functional hierarchy classification of glycan biosynthesis and metabolism, lipopolysaccharide cholinephosphotransferase and fructoselysine 6-phosphate deglycase.

**Table 4:** EggNOG functional annotations of *L. paracasei* (GMNL-653).

**Functional categories**	**Predicted gene name**	**eggNOG annot**	**KEGG brite**
Carbohydrate transport and metabolism (G)	SACC	Sucrose-6-phosphate hydrolase	Starch and sucrose metabolism
	FRYC	PTS System	Fructose-specific II-like component
	GLXK	Glycerate kinase	Biosynthesis of secondary metabolites
	AMYA	Sucrose phosphorylase	Starch and sucrose metabolism
	YHFW	Phosphotransfer between the C1 and C5 carbon atoms of pentose (By similarity)	Pentose phosphate pathway/Purine metabolism
	MALH	Glycoside hydrolase family 4	Starch and sucrose metabolism
	SP_2163	PTS system	
	GATC	PTS system, galactitol-specific IIc component	Mitochondrial DNA transcription and translation factors
	SP_2142	ROK family	
	GMUD	Glycosyl hydrolase family 1	Starch and sucrose metabolism
	FRVA	PTS system	Fructose-like PTS system EIIA component
	MANP	PTS system	
	FRLD	Sugar kinase	Fructoselysine 6-kinase
Cell wall/ membrane/ envelope biogenesis (M)	ELI_1307	Phage tail tape measure protein	
	GLF	udp-galactopyranose mutase	Glycan biosynthesis and metabolism
	RFAB	Glycosyl transferase (Group 1 family protein)	Glycan biosynthesis and metabolism
	LSGF	Glycosy ltransferase	
	LICD3	licD family	lipopolysaccharide cholinephosphotransferase
	FRLB	Catalyzes the conversion of a range of fructosamine 6-phosphates to glucose 6-phosphate and a free amino acid (By similarity)	fructoselysine 6-phosphate deglycase

## Discussion

In present study, we observed that one *L. paracasei* GMNL-653 strain reduced LPS-stimulated IL-6 and NO production in the mouse macrophage RAW264.7. GMNL-653 increased calcium mineralization in human osteosarcoma MG63 cells mineralizing in culture and protected the OVX mice from bone loss, including increasing bone volume over tissue volume (BV/TV) and bone mineral density (BMD). Our results revealed that GMNL-653 restored ovariectomy-induced gut microbiota dysbiosis and maintained intestinal barrier integrity. Additionally, in the OVX mice, GMNL-653 treatment reduced the IL-17 and LPS levels compared with those of the vehicle control group, reduced the mRNA levels of RANKL, and increased the anti-inflammatory cytokines TGF-β and IL-10 in tibia tissue. Furthermore, we applied the whole-genome sequencing technique and comparative genomics analysis to further examine these three *L. paracasei*. Our results indicated that the genes related to carbohydrate transport/metabolism and the cell wall/membrane/envelope biogenesis of GMNL-653 are worthy of future investigation. In summary, our results showed that heat-killed GMNL-653 exhibited antiosteoporotic activity through the gut microbiota–bone axis (Supplementary Figure 9).

Many studies have explored the potential role of the intestinal microbiome and its associated metabolomics in metabolic health and disease. Several metabolic diseases have been linked to altered gut microbiota, such as type 2 diabetes, cardiometabolic diseases, and non-alcoholic fatty liver disease. Disruption of gut microbiota, also called dysbiosis, is also related to multiple host disorders and is closely associated with an increased risk of bone loss (48), inflammatory bowel disease (49), diabetes, and obesity (50). Although the potential causality between various microbial components and diseases must be clarified, the intervention of probiotics or prebiotics can still provide beneficial metabolic health effects. In this study, we applied *in vitro* and *in vivo* models to validate the antiosteoporotic effect of heat-killed *L. paracasei* (GMNL-653) and employed comparative genomics analysis to explore the potentially vital genes involved.

Osteoporosis is mediated by a variety of inflammatory mediators, including TNF-α, RANKL, interleukin 1 beta, IL-6, and interferon gamma. Furthermore, chronic inflammatory disease such as inflammatory bowel disease can affect bone metabolism and is frequently associated with occurrence of osteoporosis (5, 7, 51). Although our understanding of the underlying mechanism of the relationship between inflammation and osteoporosis is incomplete, increasing evidence continues demonstrated that inflammation may intrinsically contribute to osteoporosis. Based on these findings indicating the contribution of inflammation to bone loss, we screened the anti-inflammatory activity in various strains of heat-killed probiotics in RAW 264.7 macrophages under LPS stimulation. We observed that one heat-killed *L. paracasei* GMNL-653 reduced macrophage IL-6 secretion and NO production following LPS treatment. RANKL is a key regulator of osteoclast activity and acts as a therapeutic target in osteoporosis (52). Our results indicated that the levels of *RANKL* mRNA increased in OVX mice, though subsequent treatment with heat-killed GMNL-653 once per day resulted in decreased *RANKL* mRNA expression in the tibia. Furthermore, GMNL-653 increased *TGF-*β and *IL-10* mRNA levels in the OVX mice. These results demonstrated that GMNL-653 exhibits anti-inflammatory effects, with anti-inflammatory cytokines potentially enhancing gut epithelial barrier function (53, 54) and proinflammatory cytokines attenuating intestinal barrier dysfunction (55).

IL-17A is also a vital mediator of bone absorption in inflammatory diseases associated with osteoporosis, and elevated serum concentrations of IL-17A have been observed in patients with postmenopausal osteoporosis (56). We determined that elevated IL-17A was downregulated through GMNL-653 treatment in OVX mice and that IL-17 can also promote osteoclast differentiation and RANKL secretion. Our results indicated that elevated IL-17 may enhance RANKL expression in OVX mice. Blocking the IL-17A pathway may have a positive effect on bone and cartilage damage in inflammatory disease (57). Furthermore, GMNL-653 may reduce IL-17A production, preventing bone loss in OVX mice. In terms of the influence of GMNL-653 osteoblast and osteoclast differentiation, we discovered that GMNL-653 does not influence osteoclast differentiation under RANKL stimulation. These results demonstrated that GMNL-653 can promote osteoblast differentiation leading to mineralization. The use of the OVX mice model verified that the GMNL-653 protects mice from ovariectomy-induced bone loss.

In the OVX mice, gut integrity was also altered, leading to elevated LPS in the sera. Notably, the gut microbiota composition changed in the OVX group. The PCoA plot of our beta diversity analysis indicated that the microbial communities in the OVX mice exhibited a significantly distinct cluster separate from the sham and OVX + GMNL-653 groups. Accumulated evidence revealed that the gut microbiome was associated with specific dysbiosis in osteoporosis, though the causal mechanism remains unclear. Epidemiological study also reported that the gut microbiome was altered in postmenopausal women with osteoporosis and osteopenia (58). A potential mechanism is that dysbiosis of gut microbiota may increase the permeability of the intestinal cell, increasing LPS levels in the circulation system. LPS can upregulate the inflammatory mediators in bone tissue (59). LPS has identified as the vital factor in inflammatory-induced osteoclast differentiation leading to bone loss (60, 61). Levels of sera IL-17A and LPS correlated with certain gut microorganisms in the OVX mice, indicating a link between immune responses and changes in gut microbiome composition. In addition, gut microbiota may be involved in the regulation of IL-17A production and functions (62). Therefore, we speculated that GMNL influences gut microbiota, leading to decreased IL-17A and LPS production in OVX mice.

The enriched *Bifidobacteriales* (order), *Rikenellaceae* (family), *Bifidobacteriaceae* (family), and *Bifidobacterium* spp. in the OVX + GMNL653 group exhibited significantly negative correlations with sera IL-17A. GMNL-653 intervention can enhance the abundance of these microorganisms, resulting in decreased IL-17A production. Our results demonstrated that GMNL-653 modulated gut microbiota composition in OVX mice. This result implied that the interactions of *Rikenellaceae* (family), *Bifidobacteriales* (order), *Bifidobacteriaceae* (family), *Bifidobacterium spp*. may be highly associated with IL-17. These subset of gut microbiota may have selective effects on IL-17. Previous study reported that *Bifidobacterium* suppress IL-17 production in murine splenocytes and dextran sodium sulfate-induced intestinal inflammation (63). However, whether the alterations in the abundances of *Bifidobacterium* and *Rikenellaceae* contribute to the functional consequence on the homeostasis of osteoblast and osteoclast development are issues that need further clarified. Our finding is consistent with a study reporting that gut *Rikenellaceae* involved in Th17 development (64). The enriched *Tenericutes* (phylum) and *Mollicutes* (class) in OVX group have positive correlations with sera LPS. *Tenericutes* has been reported as one of biomarkers in rat model of senile osteoporosis (65). Our result indicated that enriched *Tenericutes* was highly associated with gut barrier integrity which may result from intestinal inflammations. We further investigated the effect of GMNL-653 on the functional profiles of microbiome communities in the OVX mice and used PICRUSt to predict metagenomes mapped to the KEGG database. Carbohydrate digestion and absorption, fatty acid biosynthesis, ion channels, nitrogen metabolism, other ion-coupled transporters, pathways in cancer, phenylalanine, tyrosine, and tryptophan biosynthesis, and pyruvate metabolism were all downregulated in the OXV group compared with those in the sham group but were upregulated following GMNL-653 treatment. Several pathways have been associated with bone homeostasis; for example, essential fatty acids have been demonstrated to increase calcium absorption from the gut, reducing the urinary excretion of calcium, enhancing calcium deposition in bones, and improving bone strength (66). The dysfunction of various of ion channels is connected to perturbations in osteoblast bone formation and osteoclast bone resorption (67). Hence, ion channels play critical roles in sustaining bone homeostasis. A cohort study reported that amino acid metabolism, such as tyrosine and tryptophan metabolism, was significantly related to gut dysbiosis and osteoporosis (68). Pyruvate also plays a pivotal role in various physiological processes as a counterion and could affect the absorption and availability of calcium through the epithelial cells (69).

Our comparative genomics analysis classified genes of GMNL-653 dependent on functional categories to explore potential vital genes involved in anti-osteoporotic effects. Scanning electron microscope analysis showed that GMNL-653 has a unique longer rod-shape which may result from multicell aggregates. The carbohydrate structure and components influence bacterial adhesion and colonization. Bacterial glycans are typically display at the cell surface and interact with environment, so they have significant biomedical importance. Moreover, cell wall components are key mediators influence on the interactions between host and other microbiota. *Lacticaseibacillus* not only utilize and metabolize a variety of carbohydrates but also synthesized extensive exopolysaccharides which may affect other commensal bacteria resulting in health benefits in host. Therefore, we focus on genes exclusive express in GMNL-653 categorized as carbohydrate transport and metabolism and cell wall/membrane/envelope biogenesis. Nineteen unique genes were responsible for carbohydrate transport and metabolism and cell wall/membrane/envelope biogenesis in GMNL-653. Four genes (SACC, AMYA, MALH, and GMUD) involved in starch and sucrose metabolism. SACC, annotated as sucrose-6-phosphate hydrolase, participate in glucose 6-phosphate and fructose production. Fructooligosaccharides (FOS) are polymers of fructose with a prebiotic activity (70) which may facilitate maintaining gut barrier integrity. It was reported that FOS from *Stevia rebaudiana* roots enhanced the growth of specific strains of both *Bifidobacteria* and *Lactobacilli* (70, 71). AMYA gene encode a sucrose phosphorylase, which is a glucosyltransferase transferring glucose from sucrose to acceptor molecules further production of glucose-1-phosphate and fructose (72). The MALH gene was annotated as glycoside hydrolase, which allows the utilization of different oligosaccharides with prebiotic properties (73). Meanwhile, FRYC and FRVA gene were annotated as fructose-specific II-like component and Fructose-like PTS system EIIA component. FRLD gene classified as fructoselysine 6-kinase, which catalyzed the ATP-dependent phosphorylation of fructoselysine to fructoselysine 6-phosphate. The function of FRLB catalyze the conversion of fructoselysine to fructoselysine 6-phosphate and lysine. Lysine has been reported to enhance osteoblast proliferation, Ca^2+^ absorption and healing of bone fracture (74, 75). Therefore, GMNL-653 has sophisticated and complex genes participated in metabolisms of sucrose and fructose which may play important roles in maintaining gut barrier, symbiosis and bone metabolism.

The genes of GLF and RFAB were classified in glycan biosynthesis and metabolism. The predicted gene GLF of GMNL-653 was annotated as udp-galactopyranose mutase, which is involved in the biosynthesis of the cell wall residue of d-galactofuranose (76). The RFAB and LSGF genes were predicated as glycosyl transferase, which is responsible for exopolysaccharide production, cell aggregation, and bile resistance in *Lactobacillus* probiotic strains (77). We proposed that several bacterial cell wall components, such as lipoteichoic acids, peptidoglycans, and exopolysaccharides, may be involved in the antiosteoporotic activity of heat-killed GMNL-653. Peptidoglycan accounts for ~90% of the cell wall component of Gram-positive bacteria and is largely composed of glycan strands and pentapeptides, which present as strain-specific for *Lactobacilli* (78). Purified peptidoglycans from *L. salivarius* Ls-33 exhibited anti-inflammatory properties through the induction of IL-10 production (79).

## Conclusion

In summary, we demonstrated that heat-killed *L. paracasei* (GMNL-653) exerts antiosteoporotic effects by restoring the gut microbiota dysbiosis in ovariectomized mice. The possible mechanism of this antiosteoporotic activity involves the reduced production of inflammatory mediators (RANKL and IL-17A) and gut microbiota dysbiosis through GMNL-653 treatment. We also clarified that fluctuation in gut microbiota is closely associated with sera IL-17A and LPS levels in OVX mice. Metagenomes mapped to the KEGG database revealed that bone homeostasis is affected by gut microbial function pathways, including carbohydrate digestion and absorption, fatty acid biosynthesis, ion channels, phenylalanine, tyrosine, and tryptophan biosynthesis, and pyruvate metabolism. Whole-genome sequencing and comparative genomics analysis revealed that genes related to carbohydrate transport and metabolism and cell wall/membrane/envelope biogenesis may be associated with antiosteoporotic activity of GMNL-653. To the best of our knowledge, this is the first study linking antiosteoporotic activity, as validated using *in vitro* and *in vivo* models, to whole-genome sequencing and identifying genes potentially involved in antiosteoporotic activity.

## Data Availability Statement

The datasets presented in this study can be found in online repositories. The names of the repository/repositories and accession number(s) can be found in the article/Supplementary Material.

## Ethics Statement

The animal study was reviewed and approved by all animal experiments and housing were conducted in accordance with protocol by IACUC laboratory animal center of China Medical University and GenMont Biotech Incorporation (Taiwan IACUC Approval No. 148 IACUC 2016-165 and Approval No. 194 GenMont Biotech Incorporation Approval No. 107004).

## Author Contributions

J-HJ and T-YL analyzed the whole genome sequence, prepare figures, and wrote manuscript. W-HT analyzed and prepare figures. L-CY designed and performed the animal experiment, analyzed data, and prepared figures. C-HC designed and performed the *in vitro* experiment, analyzed data, and prepared figures. Y-TY and C-HH analyzed the gut microbiome, prepared figures, and wrote manuscript. Y-HL contributed to the study concept, wrote manuscript, and revised the final content of the manuscript. All authors contributed to the article and approved the submitted version.

## Funding

This study was supported by grants MOST-110-2320-B-008-003 from the Ministry of Science and Technology in Taiwan.

## Conflict of Interest

Authors W-HT and C-HC are employed by GenMont Biotech Incorporation. The remaining authors declare that the research was conducted in the absence of any commercial or financial relationships that could be construed as a potential conflict of interest.

## Publisher's Note

All claims expressed in this article are solely those of the authors and do not necessarily represent those of their affiliated organizations, or those of the publisher, the editors and the reviewers. Any product that may be evaluated in this article, or claim that may be made by its manufacturer, is not guaranteed or endorsed by the publisher.

## Supplementary Material

Supplementary Figure 9 Pictorial description of heat-killed Lacticaseibacillus paracasei GMNL-653 exerts antiosteoporotic effects by restoring the gut microbiota dysbiosis in ovariectomized mice.
