# Supplementary material for: Heat-Killed Lacticaseibacillus paracasei GMNL-653 Exerts Antiosteoporotic Effects by Restoring the Gut Microbiota Dysbiosis in Ovariectomized Mice
Source: Front Nutr. 2022 Feb 4;9:804210. doi: 10.3389/fnut.2022.804210 (PMC8856183; doi:10.3389/fnut.2022.804210)
Supplement: Supplementary file 2 [file Data_Sheet_2.PDF]

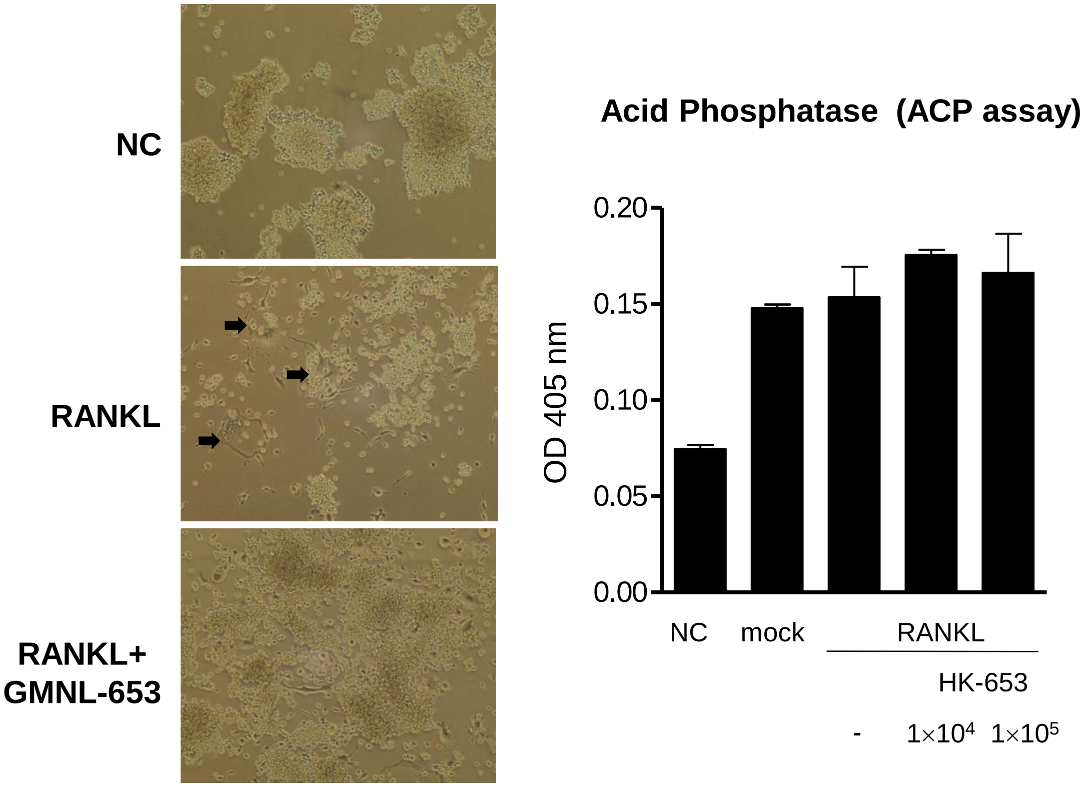

Figure S1. GMNL-653 did not influence osteoclast differentiation under RANKL stimulation. RAW264.7 cells were seeded in a 24-well plate and cultured with RANKL and GMNL-653 in α-MEM. After 7 days, the cells were stained with tartrate-resistant acid phosphate. Acid phosphatase-positive multinucleated (>3 nuclei) cells were counted as osteoclasts, and an acid phosphatase colorimetric assay was employed to measure the acid phosphatase activity.

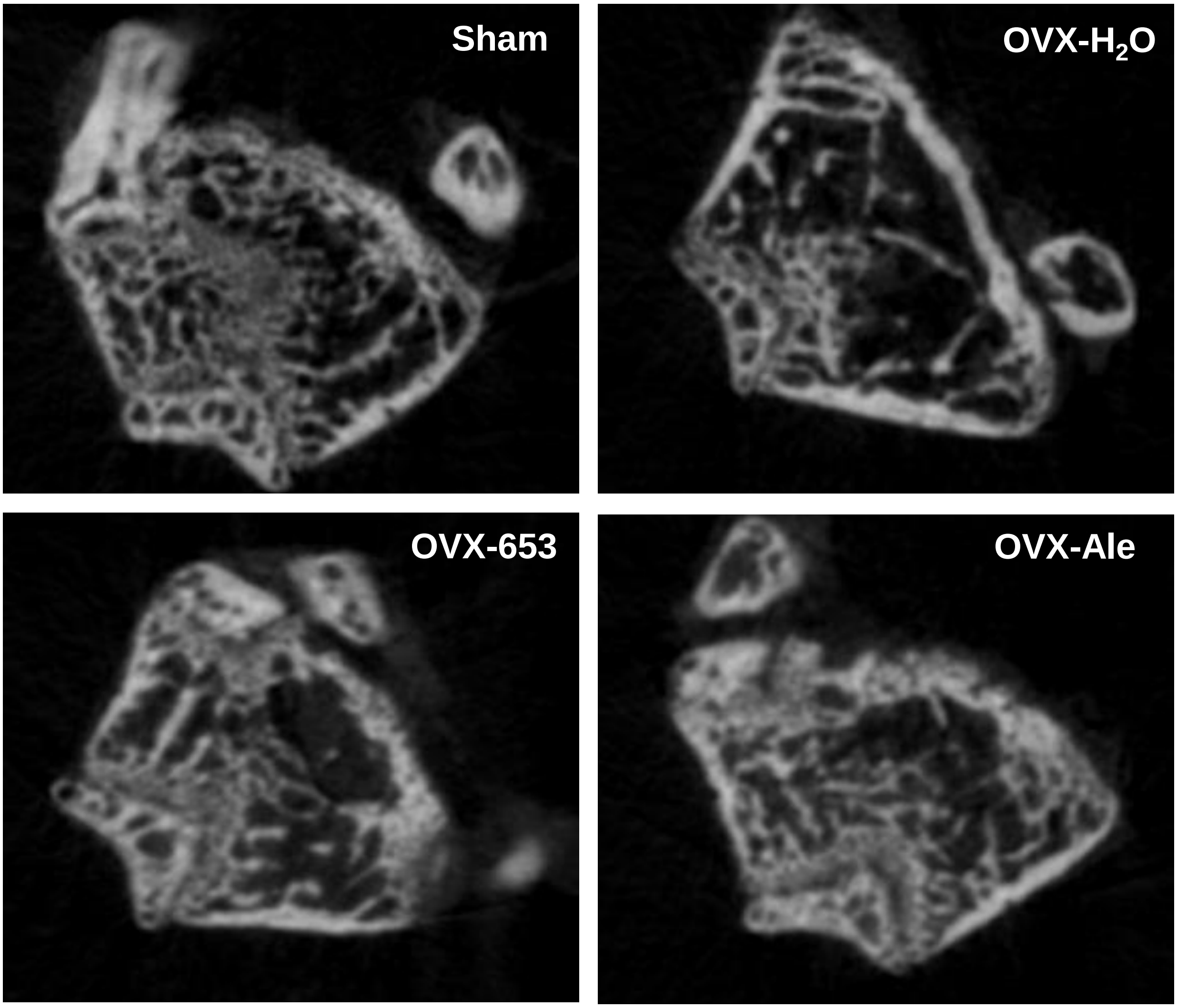

Figure S2. Micro CT analysis on bone architecture of tibia in ovariectomized (OVX) mice treated with either the vehicle (H_2O) or heat-killed *L. paracasei* (GMNL-653). OVX-Alendronate (Ale) acted as a positive control to treat and prevent osteoporosis in OVX mice.

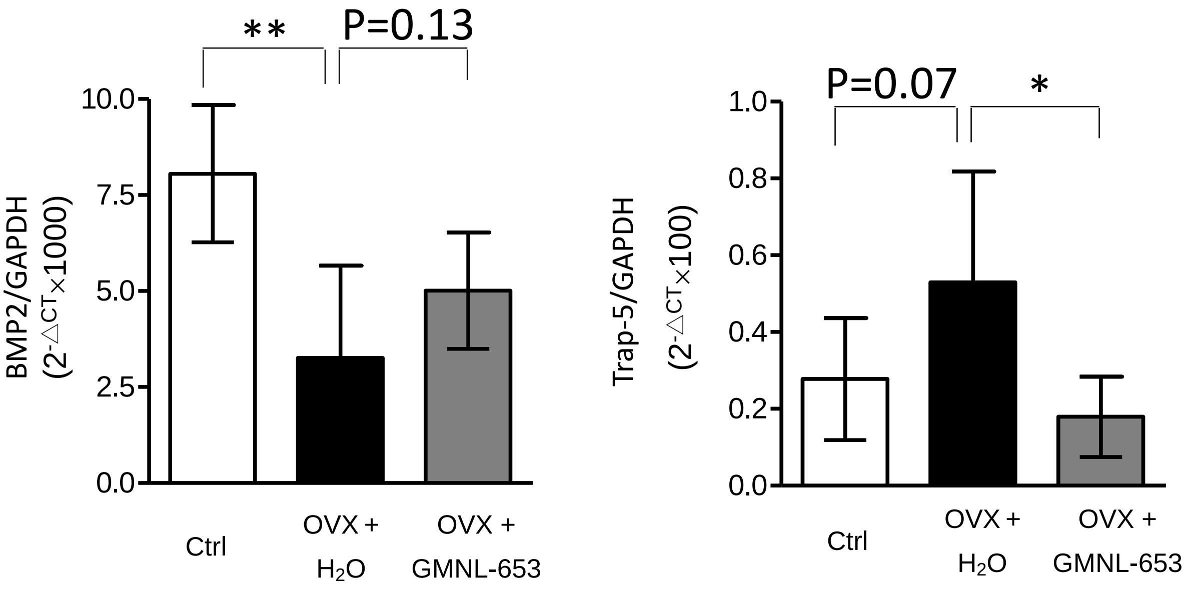

Figure S3. The mRNA levels of BMP-2 and Trap-5 in the tibia of ovariectomized (OVX) mice treated with either the vehicle (H_2O) or heat-killed L. paracasei (GMNL-653). qRT-PCR analysis of the expression of genes known to regulate osteoblast differentiation, and bone resorption and osteoclast number. The genes expression level of BMP-2 and Trap-5 were detected using following primers: forward primer 5'- AGCTGCAAGAGACACCCTTT-3' and reverse primer 5'- CATGCCTTAGGGATTTTGGA-3' for mouse BMP-2; forward primer 5'-GACGATGGGCGCTGACTTCA-3' and reverse primer 5'-GCGCTTGGAGATCTTAGAGT-3' for mouse Trap-5.

A.

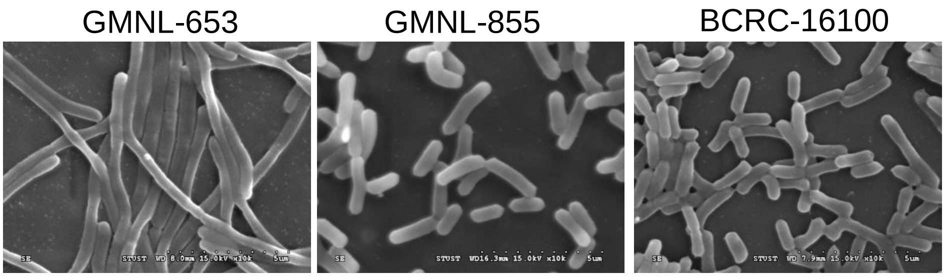

B.

strain	16S rRNA sequencing	Whole genmone sequencing
GMNL-653	*L. paracasei subsp. paracasei*	*L. paracasei*
GMNL-855	*L. paracasei subsp. paracasei*	*L. paracasei*
BCRC-16100	*L. paracasei subsp. paracasei*	*L. paracasei*

C.

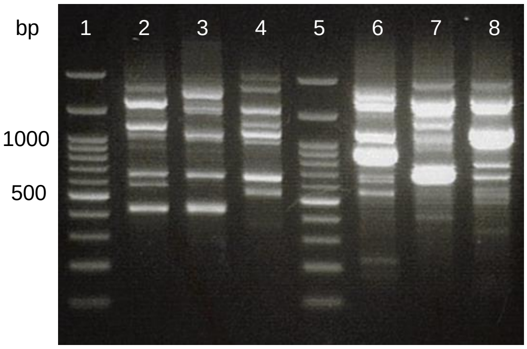

Lane1: marker
Lane2: GMNL-653 (LacP2)
Lane3: GMNL-855 (LacP2)
Lane4: BCRC-16100 (LacP2)
Lane5: marker
Lane6: GMNL-653 (LacP3)
Lane7: GMNL-855 (LacP3)
Lane8: BCRC-16100 (LacP3)

D.

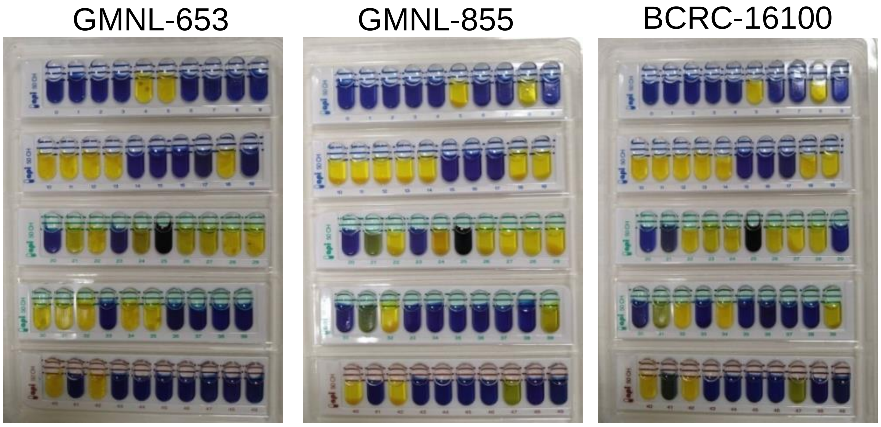

Figure S4. Identification of the three strains of *L. paracasei*, GMNL-653, GMNL-855, and BCRC-16100. (**A**) Scanning electron microscope analysis of the three strains. (**B**) 16S rRNA partial sequencing was determined using a PAF (5′-AGAGTTTGATCCTGGCTCAG-3′) and V3R primer (5′- ATC TAC GCA TTT CAC CGC TAC-3) through PCR analysis, respectively. Following NCBI BLAST analysis, GMNL-653, GMNL-855, and BCRC-16100 were determined to be *L. paracasei* strains. (**C**) RAPD analysis of the three strains was conducted using LacP2 (5′-ACGCGCCCT) or LaCP3(5′- CCGCAGCGTT) primers. The results demonstrated that they are differential strains.

Strips no.	carbohydrates substrate	**GMNL-653**	**GMNL-855**	**BCRC-16100**
1	Glycerol	-	-	-
2	Erythritol	-	-	-
3	D-Arabinose	-	-	-
4	L-Arabinose	+	-	-
5	D-Ribose	+	+	+
6	D-Xylose	-	-	-
7	L-Xylose	-	-	-
8	D-Adonitol	-	+	+
9	Methyl-β-D-Xylopyranoside	-	-	-
10	D-Galactose	+	+	+
11	D-Glucose	+	+	+
12	D-Fructose	+	+	+
13	D-Mannose	+	+	+
14	L-Sorbose	-	+	+
15	L-Rhamnose	-	-	-
16	Dulcitol	-	-	-
17	Inositol	-	-	-
18	D-Mannitol	+	+	+
19	D-Sorbitol	-	+	+
20	Methyl-α-D-mannopyranoside	-	-	-
21	Methyl-α-D-glucopyranoside	+	-	-
22	N-Acetyl glucosamine	+	+	+
23	Amygdalin	-	-	+
24	Arbutin	+	+	+
25	Esculin ferric citrate	+	+	+
26	Salicin	+	+	+
27	D-Cellobiose	+	+	+
28	D-Maltose	+	+	+
29	D-Lactose (bovine origin)	+	+	-
30	D-Melibiose	+	-	-
31	D-Saccharose (sucrose)	+	-	-
32	D-Trehalose	+	+	+
33	Inulin	-	-	-
34	D-Melezitose	+	-	+
35	D-Raffinose	+	-	-
36	Amidon (starch)	-	-	-
37	Glycogen	-	-	-
38	Xylitol	-	-	-
39	Gentiobiose	-	+	+
40	D-Turanose	+	+	+
41	D-Lyxose	-	-	-
42	D-Tagatose	+	+	+
43	D-Fucose	-	-	-
44	L-Fucose	-	-	-
45	D-Arabitol	-	-	-
46	L-Arabitol	-	-	-
47	Potassium gluconate	-	-	-
48	Potassium 2-ketogluconate	-	-	-
49	Potassium 5-ketogluconate	-	-	-
-: negative; +: positive				
W: weak positive reaction				

Figure S4. (cont'd) Carbohydrate fermentation ability was assessed using API 50 CHL to identify *Lactobacillus* and related genera. The carbohydrate activity patterns of three strains were summarized.

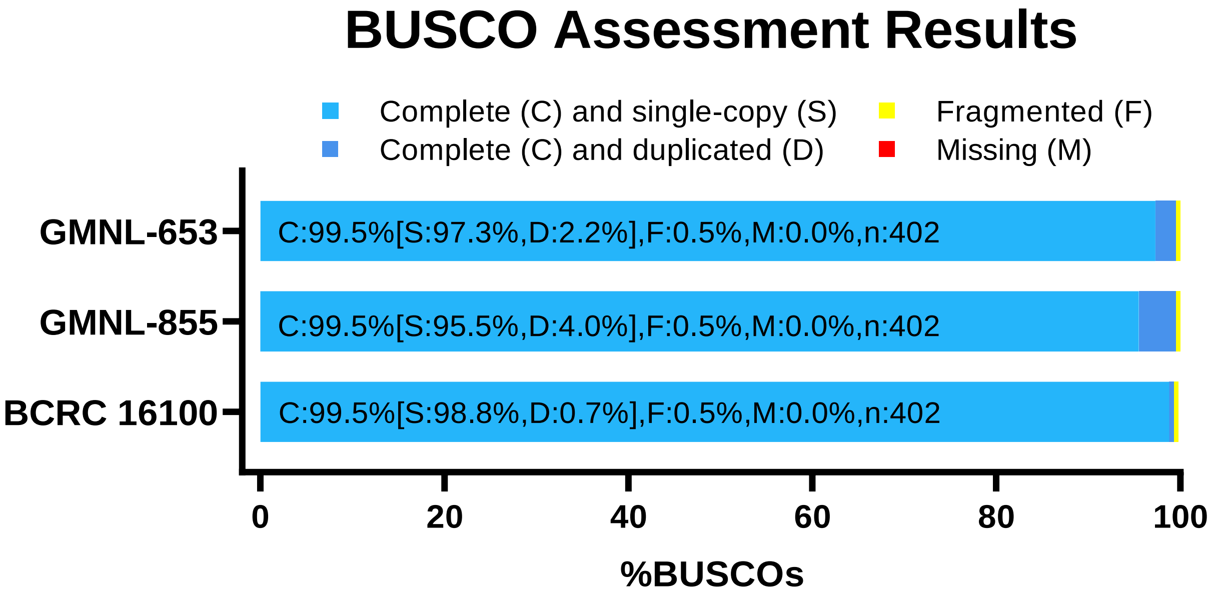

Figure S5. BUSCO completeness assessments for genomics data quality control. Bar charts produced with the BUSCO plotting tool show proportions classified as complete (C, blues), complete single-copy (S, light blue), complete duplicated (D, dark blue), fragmented (F, yellow), and missing (M, red).

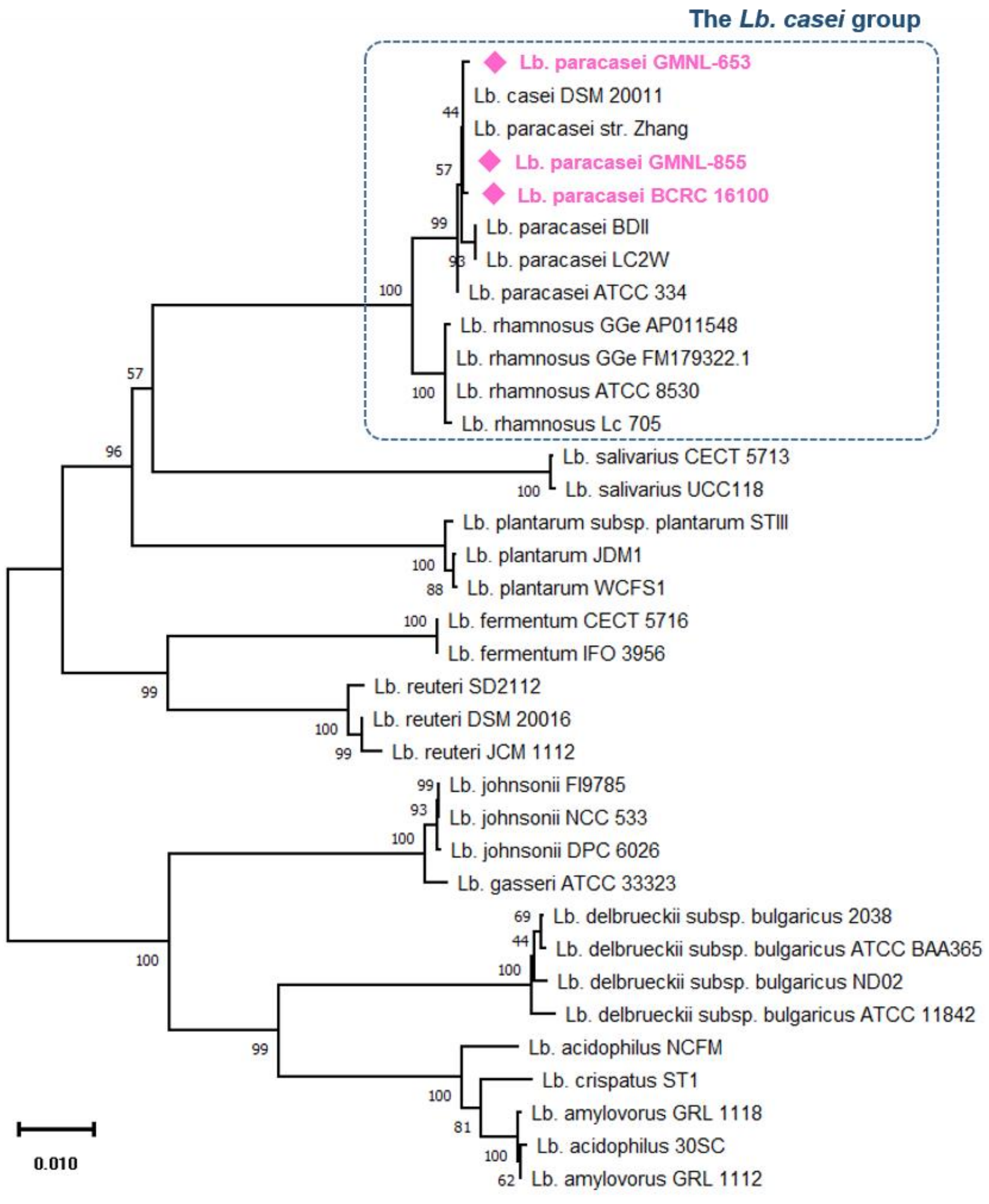

Figure S6. Molecular phylogenetic analysis of *the L. paracasei* strain using the maximum-likelihood estimation method based on the Kimura two-parameter model to infer the evolutionary history. The tree with the highest log likelihood is indicated. The percentage of trees in which the associated taxa are clustered together is presented next to the branches. The initial tree(s) for the heuristic search was obtained automatically through the application of the neighbor-joining and BIONJ algorithms to a matrix of pairwise distances estimated using the maximum composite likelihood approach; the topology with the superior log likelihood value was then selected. The tree is drawn to scale, with branch lengths representing the number of substitutions per site. The analysis involved 35 nucleotide sequences, with a total of 1,660 positions in the final dataset. Evolutionary analyses were conducted using MEGA X.

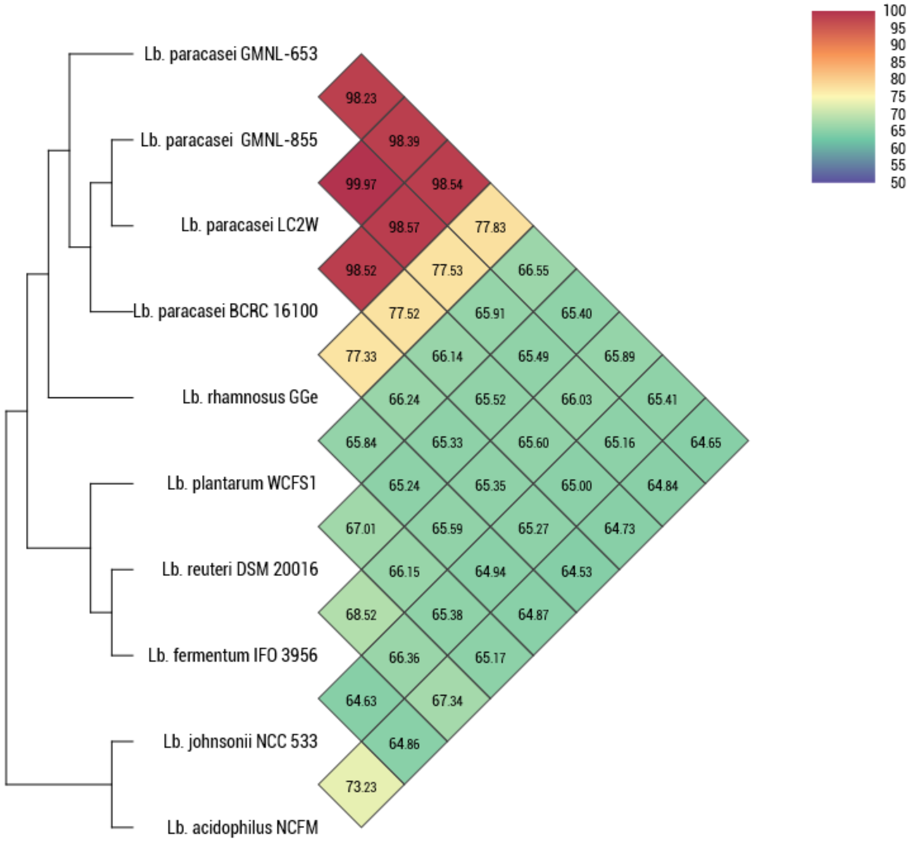

Figure S7. Heat-map and phylogenetic trees based on average nucleotide identity values determined for *L. paracasei* and related strains.

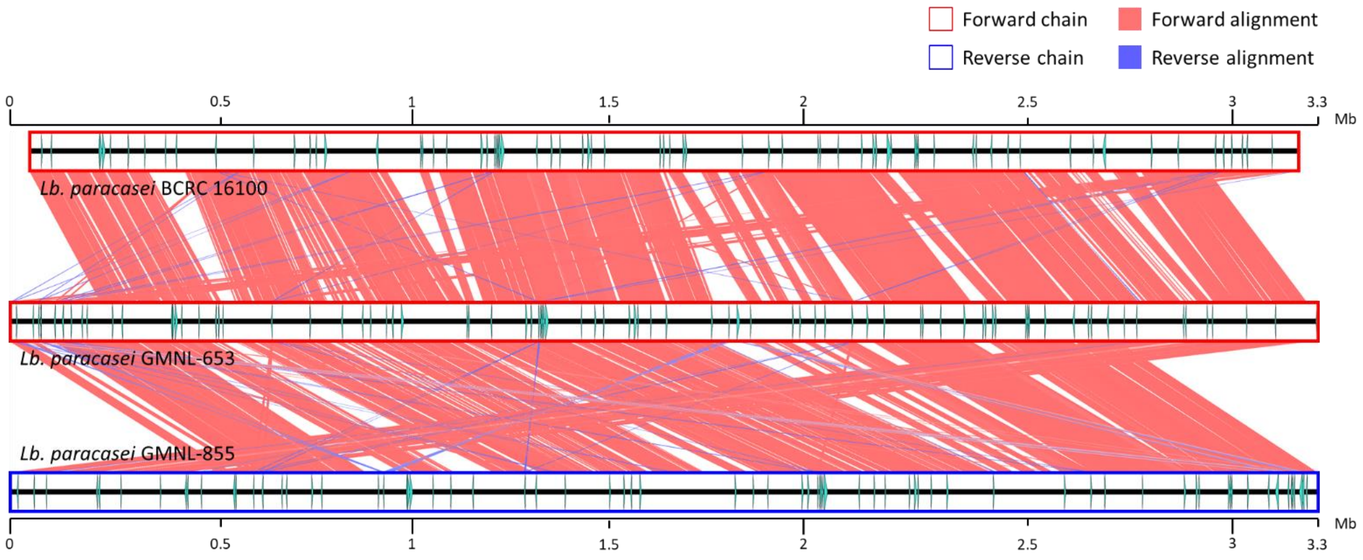

Figure S8. Genome-wide comparison between *L. paracasei* GMNL-653 ,GMNL-855 and BCRC 16100 at the chromosome region by Easyfig genome comparison visualizer.
