# Supplementary figures and images for: Heat-Killed Lacticaseibacillus paracasei GMNL-653 Exerts Antiosteoporotic Effects by Restoring the Gut Microbiota Dysbiosis in Ovariectomized Mice
Source: Front Nutr. 2022 Feb 4;9:804210. doi: 10.3389/fnut.2022.804210 (PMC8856183; doi:10.3389/fnut.2022.804210)

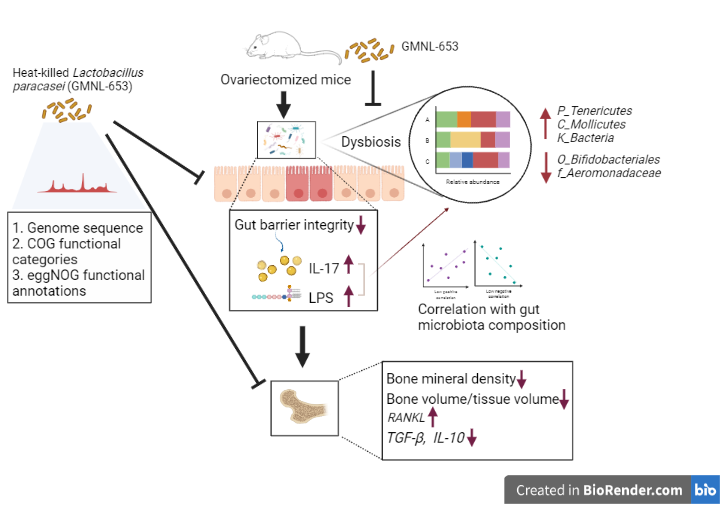

Supplement: Supplementary Figure 9 — Pictorial description of heat-killed Lacticaseibacillus paracasei GMNL-653 exerts antiosteoporotic effects by restoring the gut microbiota dysbiosis in ovariectomized mice. [file Image_9.TIF]
